# The Hyperporphyrin Concept: A Contemporary Perspective

**DOI:** 10.1021/jacsau.2c00255

**Published:** 2022-06-30

**Authors:** Carl C. Wamser, Abhik Ghosh

**Affiliations:** † Department of Chemistry, Portland State University, Portland, Oregon 97207-0751, United States; ‡ Department of Chemistry and Arctic Center for Sustainable Energy, UiT − The Arctic University of Norway, N-9037 Tromsø, Norway

**Keywords:** hyperporphyrin, hypsoporphyrin, hypercorrole, hypsocorrole, Gouterman four-orbital model

## Abstract

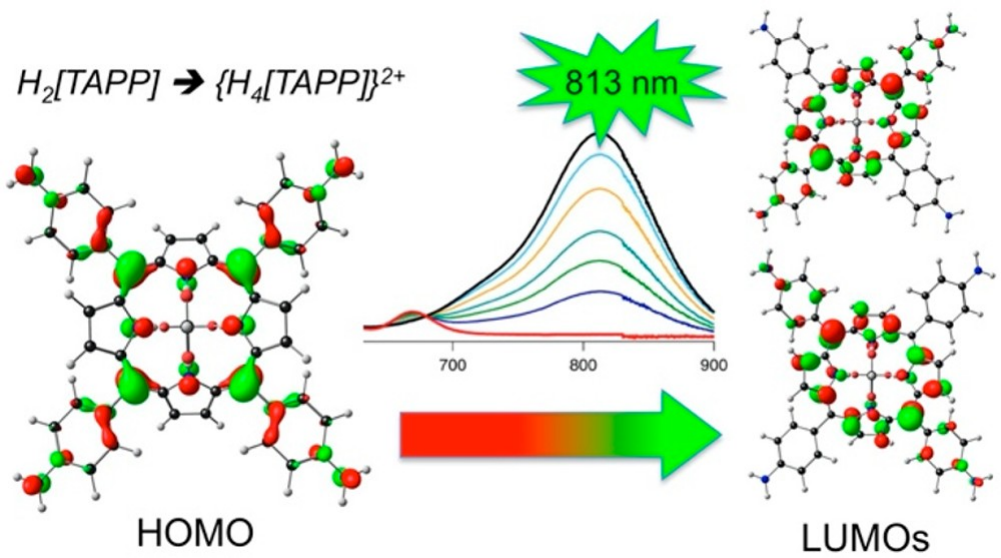

The Gouterman four-orbital model conceptualizes porphyrin UV–visible spectra as dominated by four frontier molecular orbitals—two nearly degenerate HOMOs and two exactly degenerate LUMOS under *D*_4h_ symmetry. These are well separated from all the other molecular orbitals, and normal spectra involve transitions among these MOs. Unusual spectra occur when additional orbitals appear in this energy range, typically as a consequence of the central coordinated atom. For example, metals with empty d orbitals in a suitable energy range may lead to charge transfer from porphyrin (ligand) to metal, that is, so-called LMCT transitions. Metals with filled p or d orbitals may lead to charge transfer from metal to porphyrin, MLCT transitions. These cases lead to additional peaks and/or significant redshifts in the spectra and were classified as hyperporphyrins by Gouterman. Cases in which spectra are blueshifted were classified as hypsoporphyrins; they are common for relatively electronegative late transition metal porphyrins. Many of the same principles apply to porphyrin analogues, especially corroles. In this Perspective, we focus on two newer classes of hyperporphyrins: one reflecting substituent effects in protonated or deprotonated free-base tetraphenyporphyrins and the other reflecting “noninnocent” interactions between central metal ions and corroles. Hyperporphyrin effects on spectra can be dramatic, yet they can be generated by relatively simple changes and subtle structural variations, such as acid–base reactions or the selection of a central metal ion. These concepts suggest strategies for engineering porphyrin or porphyrinoid dyes for specific applications, especially those requiring far-red or near-infrared absorption or emission.

## 1 Introduction

Porphyrins and their analogues exhibit a wide range of properties that underlie a myriad of biological roles and an ever-expanding number of applications in chemistry, medicine, and the technological sphere. These have been documented in numerous review articles and the ongoing book series *Handbook of Porphyrin Science*, now running to 45 volumes with 217 chapters,^1^ as well as in numerous review articles (one of the most notable being a special issue of *Chemical Reviews* on expanded, contracted, and isomeric porphyrins^200^). A distinctive hallmark of porphyrin-type macrocycles is their rich array of optical properties, which are also reflected in a correspondingly wide range of electronic properties. Furthermore, simple substituent-mediated tuning of optical and electronic properties allows for facile application in chemical catalysis, photocatalysis, medicine, and increasingly, materials science and technology. This Perspective aims to summarize and further elucidate the mechanisms underlying the tunability of the optical and electronic properties of porphyrins, specifically the generation and characterization of hyperporphyrins.

**Figure 1:**
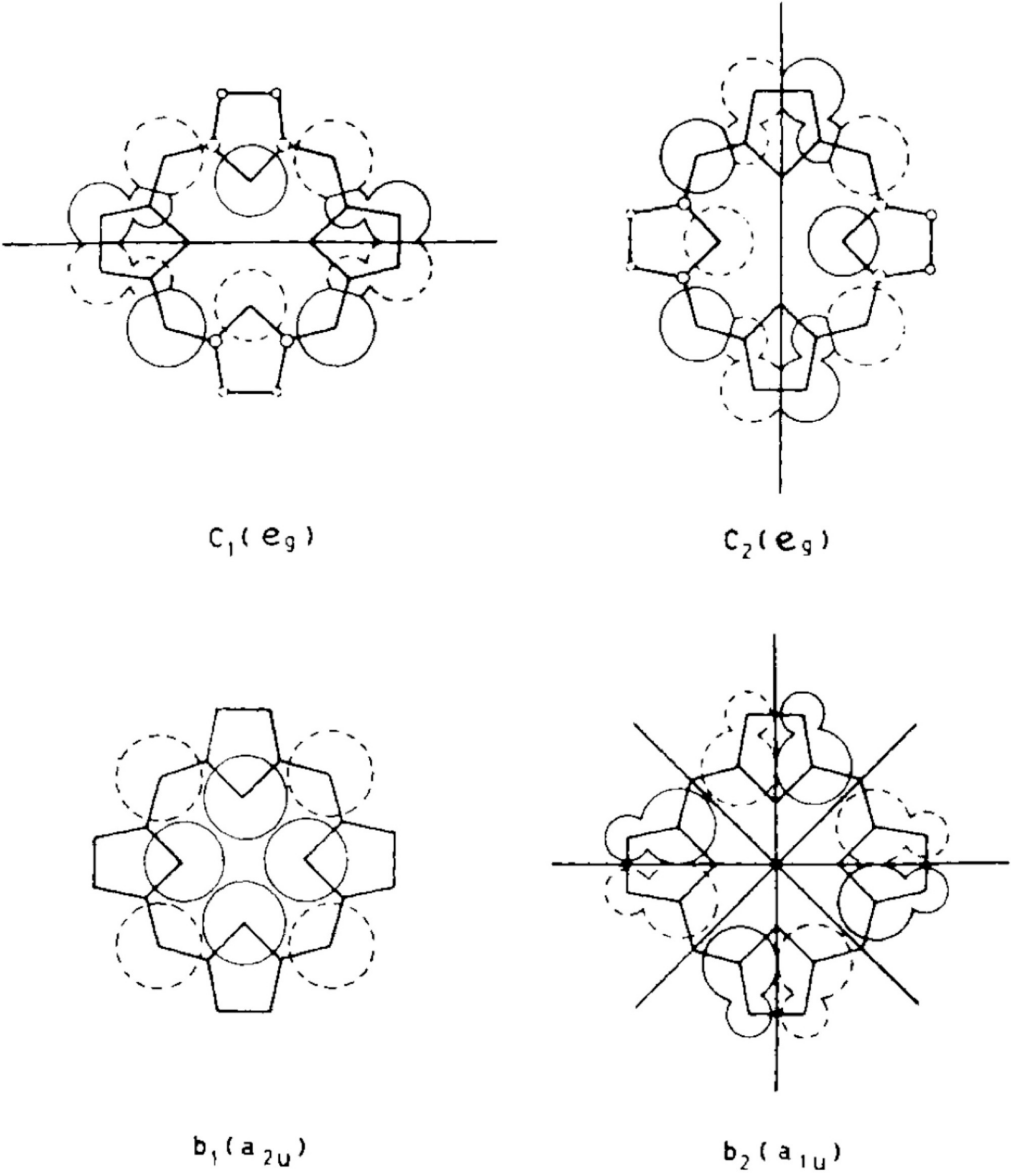
Gouterman’s historic diagram of porphyrin MOs. The atomic orbital coefficients are proportional to the size of the circles; solid or dashed circles indicate sign. Symmetry nodes are drawn in heavy lines. Reproduced with permission from ref (3). Copyright 1961 Elsevier.

Martin Gouterman,^2^ who passed away recently, is widely regarded as the father of modern porphyrin spectroscopy and introduced the term hyperporphyrin. He is arguably best remembered for his eponymous four-orbital model, which was developed in the early 1960s on the basis of extended Hückel calculations.^3,4^ The calculations on the porphyrin π system identified two HOMOs and two LUMOs energetically well separated from all other π molecular orbitals: the two HOMOs are nearly degenerate (a_1u_ and a_2u_ under *D*_4h_ symmetry), and the two LUMOs are exactly degenerate (e_g_ under *D*_4h_). Configuration interaction was taken into account, and the characteristic porphyrin Q and B (Soret) bands were accurately assigned to transitions among these four molecular orbitals (Figure 1). Simple perturbation theory arguments then explained why blood (i.e., hemoglobin) is red and grass (i.e., chlorophyll) is green.

Subsequently, Gouterman embarked on a systematic survey of porphyrins and structural analogues, in which he made good use of new compounds synthesized by Buchler, Dolphin, and Adler, among others. In 1978, he presented a masterful optical taxonomy of porphyrins on the basis of their absorption and emission properties.^5^ He distinguished three broad classes of porphyrins: normal, hypso, and hyper. These terms are used to describe both the porphyrins and their spectra.

Normal porphyrin spectra refer to those observed for free-base and closed-shell metal (e.g., Mg and Zn) derivatives of simple porphyrins such as tetraphenyl- or octaethylporphyrin. These spectra show the classic Q and B bands, as well as an N band in the near-UV, and are generally well described by the four-orbital model. Early in the development of corrole chemistry, it was established that simple closed-shell metallocorroles, such as axially coordinated Al^6^ and Ga^7,8^ corroles, also conform to the four-orbital model^9^ and exhibit so-called “normal” spectra.

Hypsoporphyrin spectra are similar to normal spectra but with blueshifted Soret and Q bands, exemplified by late transition metal porphyrins involving such elements as Co, Ni, Pd, and Cu. The blueshifts were long thought to reflect backbonding-induced elevation of the porphyrin e_g_ LUMOs.^5^ A recent reinvestigation, however, ascribes the hypso effect to lower a_2u_ HOMO levels in metalloporphyrins with less electropositive metal centers.^10^

In contrast, hyperporphyrin spectra show bands that are redshifted relative to normal spectra and, in particular, are defined as showing “prominent extra absorption bands in addition to Q, B, and N in the region λ > 320 nm.”^5^ Gouterman went on to distinguish between two major classes of hyperporphyrins, the p-type and the d-type:

“(1) p-Type hyperporphyrins are found with main group metals in lower oxidation states, that is, Sn(II), Pb(II), As(III), Sb(III), and Bi(III). The extra bands are fairly well established as due to charge transfer (CT) transitions a_2u_ (np_z_) (metal) → e_g_(π*) (ring).

“(2) d-Type hyperporphyrins are found with transition metals in configurations d^m^, 1 < *m* < 6, that have holes in the e_g_(d_π_) orbitals and relatively stable lower oxidation states. The extra bands, with somewhat less certainty, are attributed to CT transitions a_lu_ (π), a_2u_ (π) (ring) → e_g_(d_π_) (metal).”^5^

In general, the unique features of metalloporphyrin hyper spectra reflect the presence of additional orbitals in the vicinity of the four porphyrin frontier orbitals. If a central metal (or element) possesses filled valence p orbitals of appropriate symmetry, a metal-to-ligand charge transfer (MLCT) transition is typically observed. However, if the metal harbors empty d orbitals of appropriate symmetry, a ligand-to-metal charge transfer (LMCT) transition is often observed. In both cases, the new transition will necessarily be of lower energy (redshifted) compared with what is predicted by the four-orbital model. The observation and interpretation of hyper spectra led to the correct identification of a number of key heme protein intermediates, especially for thiolate-ligated heme proteins such as chloroperoxidase and cytochrome P450.^11^

The present Perspective is not intended to be a comprehensive survey of hyperporphyrins. Instead, after presenting a short introduction to p- and d-type hyperporphyrins, we will focus on two avenues of research on hyperporphyrin systems that we have pursued in recent years in our own laboratories. The first of these, originating largely from the Wamser laboratory, centers around protonated free-base *meso*-tetraarylporphyrins, for which the redshifted spectral features are thought to reflect aryl-to-porphyrin, i.e., ligand-to-ligand, charge transfer (LLCT) transitions. The second line of work, originating largely from the Ghosh laboratory, focuses on certain *meso*-triarylcorrole derivatives, in which similar aryl-to-corrole LLCT transitions account for strong substituent effects on the position of the Soret maxima.

## 2 Classical p- and d-Type Hyperporphyrins

As mentioned above, p-type hyperporphyrin systems typically involve lower-valent p-block element centers with a lone pair. Classic p-type hyperporphyrins include trivalent Group 13 (As, Sb, and Bi) and divalent Group 14 (Ge, Sn, and Pb) porphyrins.^12^ Although the first examples were synthesized almost a century ago, some of the classic synthetic work was reported by Johann Buchler,^13^ who provided samples to Gouterman for detailed spectroscopic studies. The unsung hero of the field was Phil Sayer, who was a doctoral student of Gouterman and Rex Robinson, subsequently a postdoctoral associate in Gouterman’s laboratory, and a meticulous physical chemist. Sayer’s experimental work—during the 1970s until his untimely death in 1985, while still a member of Gouterman’s laboratory—still forms much of what we know of p-type hyperporphyrin spectra.^12^

**Figure 2:**
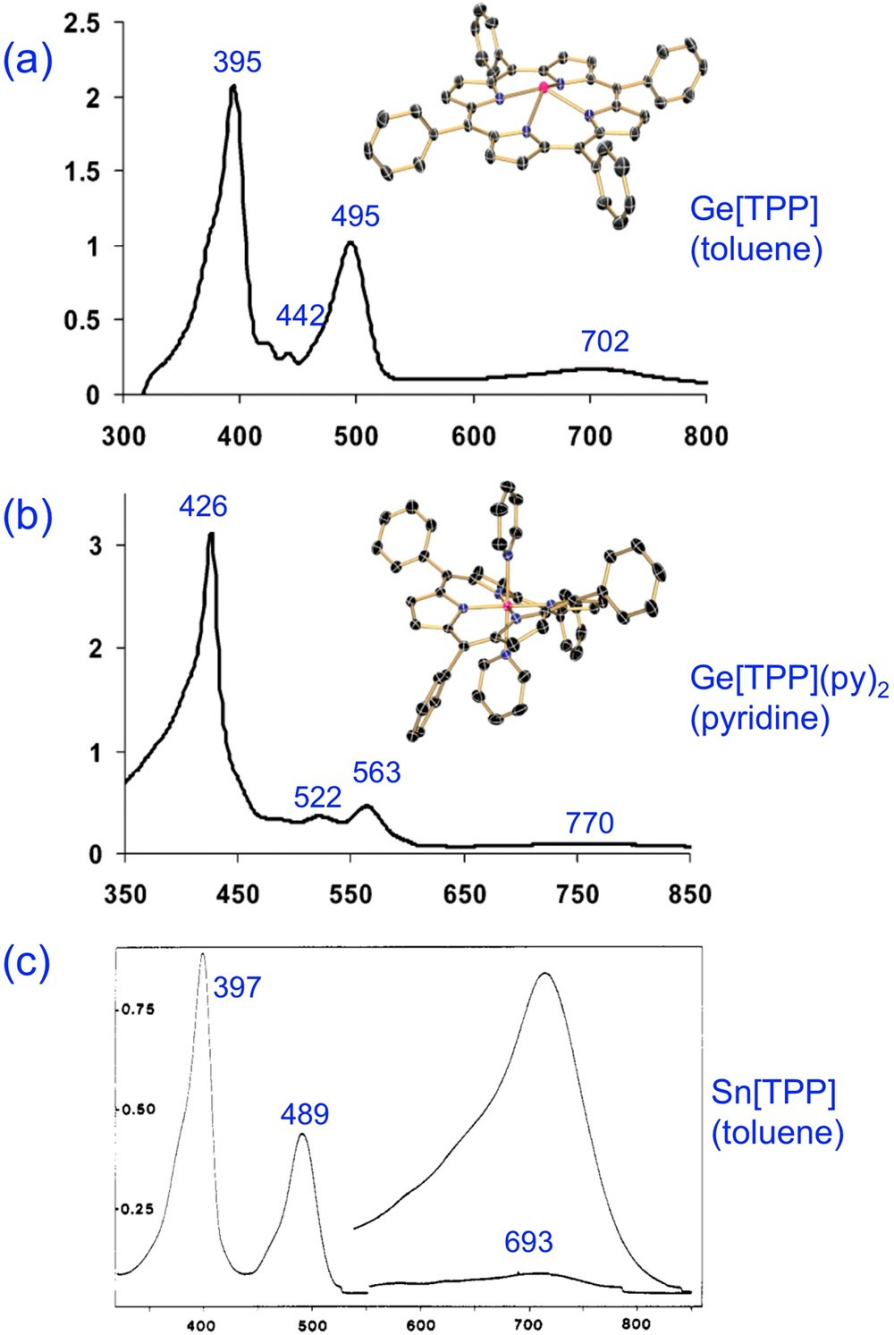
UV–vis–NIR spectra of reduced Ge and Sn porphyrins. Adapted with permission from refs^14^ and^15^. Copyright 2007 and 1990, respectively, American Chemical Society.

**Figure 3:**
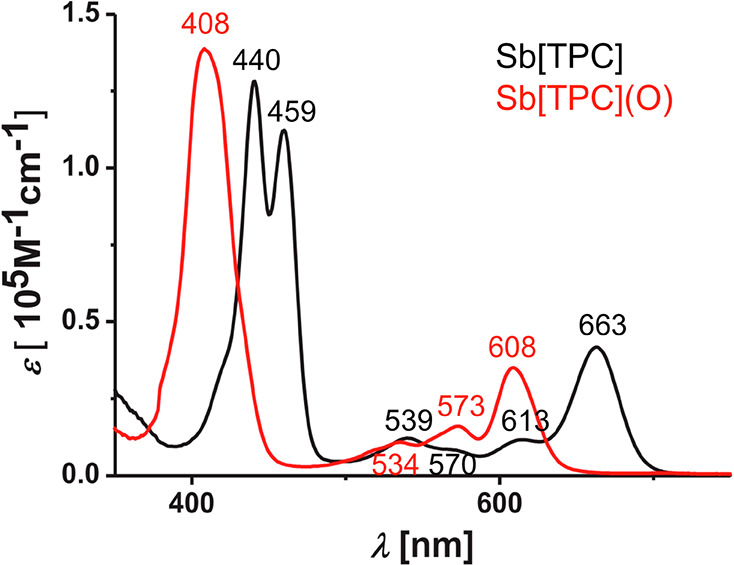
Electronic absorption spectra of Sb and SbO triphenylcorrole (TPC) derivatives. Adapted with permission from ref (19). Copyright 2020 Elsevier.

Figure 2 depicts the classic p-type hyperporphyrin spectra of divalent Ge^14^ and Sn^15,16^ tetraphenylporphyrin (TPP) derivatives. These are characterized by a split, or shouldered, Soret band and a lowest-energy Q band that extends well into the red or near-infrared. As mentioned, the extra features in these spectra are thought to involve excitations from one or more MOs with p-element lone pair character into the porphyrin LUMOs. Detailed, modern quantum chemical studies of these spectra, however, are yet to be reported. Interestingly, trivalent antimony corroles may provide a fascinating example of a p-type “hypercorrole” spectrum; oxidation to an SbO corrole results in a spectral blueshift, that is, a so-called “normal” corrole spectrum (Figure 3).^17−19^

**Chart 1:**
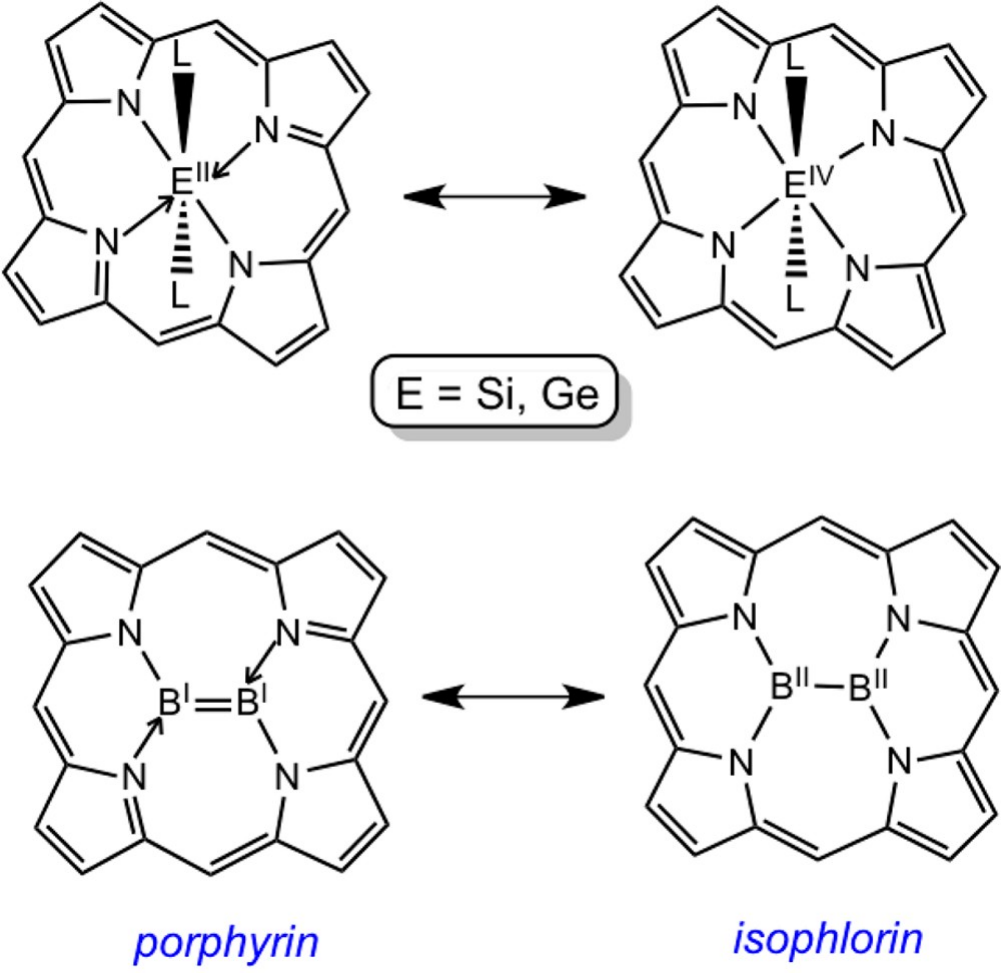
Limiting-Case Descriptions for Reduced Group 14 (E = Si, Ge) and Diboron Porphyrins, Adapted from Ref (22); Copyright 2019 American Chemical Society

A contemporary account of p-type hyperporphyrins would be incomplete without some mention of the remarkable axial ligand reactivity of germanium(II) porphyrins. Vaid et al. found that the addition of axial ligands to these complexes results in dramatic electronic-structural and spectral changes, reflecting an intramolecular redox process that effects a two-electron oxidation of the Ge and a two-electron reduction of the porphyrin to isophlorin.^14^ Subsequently, the authors reported a similar Si isophlorin system,^20^ while Brothers et al. reported an analogous diboron isophlorin.^21^ It should be noted that these systems require a careful distinction between valence and oxidation state (the latter being indicated with Roman numerals in Chart 1).^22,23^

The archetypal examples of hyperporphyrins—specifically d-type hyperporphyrins—involve middle transition metal derivatives with d electron counts less than six, such as Cr(III), Mn(III), and Fe(III) porphyrins.^5^ In contrast, d electron counts of six or higher often lead to hypsoporphyrins. Archetypal examples of the latter include Co(III), Co(II), Ni(II), Pd(II), Pt(II), and Cu(II); simple porphyrin derivatives of these metal ions typically exhibit blueshifted Soret and Q bands relative to their Mg and Zn analogues. Gouterman considered the hyper/hypso distinction to be significant because, unlike d-type hyperporphyrins, many (but not all) hypsoporphyrins exhibit characteristic emission properties.^5^ For example, Pd(II), Pt(II), and Ir(III) porphyrin derivatives are typically strongly phosphorescent. Corrole analogues of hypsoporphyrins include many 5d metallocorroles such as d^2^ Os(VI)N corroles, d^6^ Ir(III) and Pt(IV) corroles, and d^8^ Au(III) corroles, which exhibit both hypsochromically shifted Soret and Q bands as well as near-IR phosphorescence at room temperature.^24^

**Figure 4:**
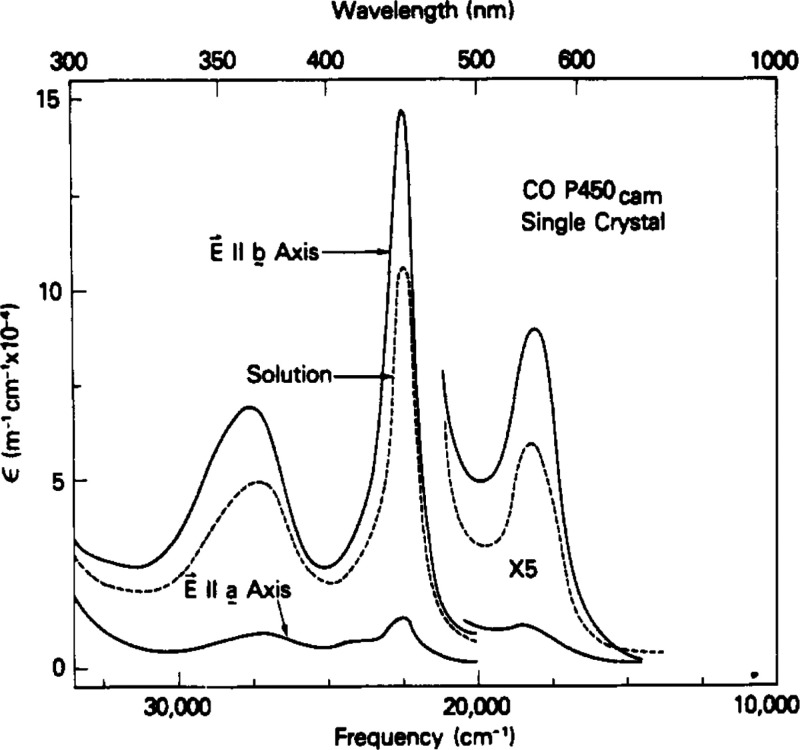
CO-P450_cam_ polarized single-crystal and solution absorption spectra. Adapted from ref (25). Copyright 1976 American Chemical Society.

**Figure 5:**
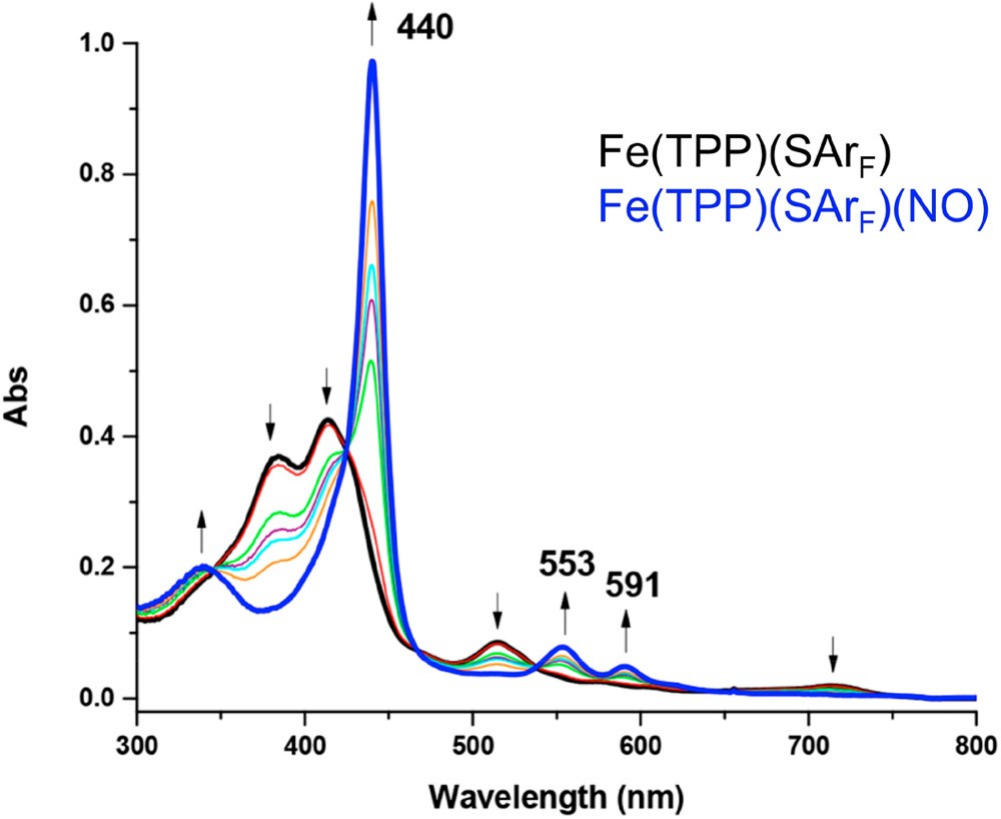
Formation of the thiolate-ligated {FeNO}^6^ (blue) via nitrosylation of the corresponding five-coordinate ferric TPP (black). Adapted from ref (26). Copyright 2019 American Chemical Society.

Iron porphyrins exemplify some of the most instructive and important examples of hyperporphyrins. We hasten to add, however, that most six-coordinate hemes as well as their Ru(II) and Os(II) analogues exhibit hypso spectra.^5^ A classic example of an iron(II) porphyrin with a hyper spectrum is provided by CO-ligated cytochrome P450. Besides a moderately redshifted Soret band at 446 nm, the enzyme was also found to exhibit a strong near-UV band at 363 nm. Interestingly, such a spectrum could also be generated by passing CO into a solution of ferro-protoheme IX and a thiolate (but not a thiol). In a polarized single-crystal UV–vis study,^25^ Hanson et al. (Gouterman’s group) established that both bands represented the full concentration of the enzyme (i.e., a single species) and also had the same polarization (Figure 4). The logical conclusion was made that a near-UV excitation of *E*_u_ symmetry was mixing heavily with a classic Soret transition to steal the latter’s intensity and push it to the red. Subsequent extended Hückel calculations clearly implicated the axial thiolate in determining the Soret and post-Soret absorption profile of P450, with a substantial amount of sulfur character mixing in with the porphyrin a_2u_ HOMO.^25^ The extra 363 nm Soret feature qualifies P450 as a hyperporphyrin, and Hanson and co-workers correctly noted an analogy with p-type hyperporphyrins, which also exhibit split Soret bands. Perhaps most notably, these studies established the heme–thiolate core of cytochrome P450. Much more recently, it has become clear that {FeNO}^6^-heme–thiolate complexes isoelectronic to cytochrome P450 also exhibit qualitatively similar split Soret features (Figure 5).^26^

Higher-valent heme protein intermediates also exhibit hyperporphyrin spectra, as illustrated by the following examples.

**Figure 6:**
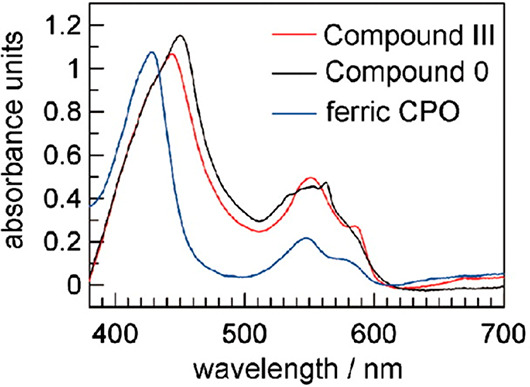
Electronic absorption of chloroperoxidase crystals, mounted in a loop and kept at 90 K: ferric ground state (blue), Compound III (red), and Compound 0 (black). See text for definitions of these states. Adapted from ref (27). Copyright 2007 National Academy of Sciences.

Figure 6 presents crystal absorption spectra of three different states of the heme–thiolate protein chloroperoxidase (from the fungus *Caldariomyces fumago*),^27^ which catalyzes hydrogen-peroxide-mediated halogenation reactions. The spectra depicted are those for the Fe(III)–thiolate resting state, the ferric hydroperoxide state (Compound 0), and a ferric superoxide or oxyheme state (Compound III).

**Figure 7:**
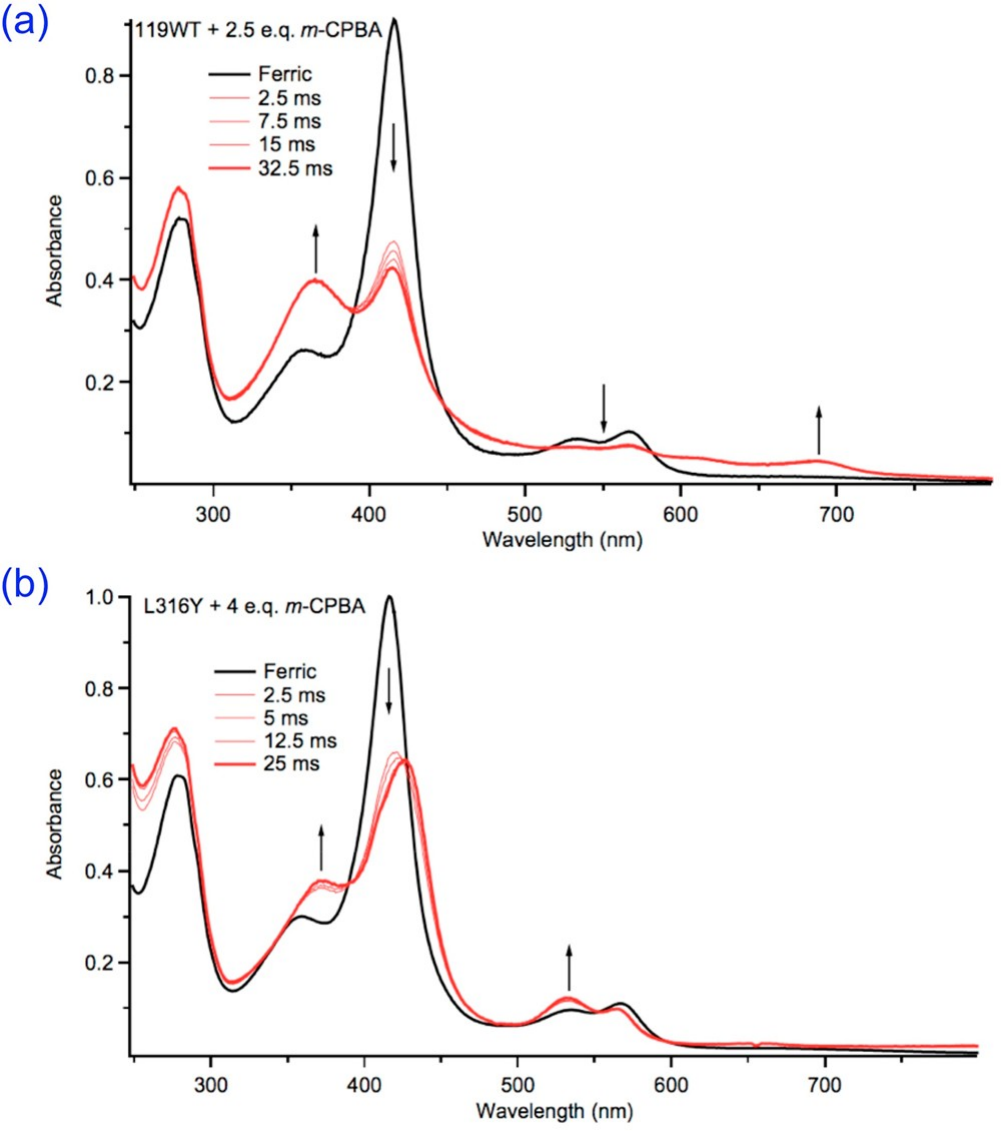
Reaction of *m*-CPBA with wild-type CYP119 generates Compound I in high yield (top). The L316Y CYP119 variant incorporates a tyrosine at the same position as Y352 in CYP158. The reaction of this variant with *m*-CPBA generates Compound II in high yield (bottom). Adapted with permission from refs (29) and (30). Copyright 2010 and 2013, respectively, American Association for the Advancement of Science.

Figure 7 depicts the electronic absorption spectra of Compound I and Compound II, which are high-valent Fe(IV) states of the P450 enzyme CYP119.^28−31^ Both states exhibit distinctive split Soret bands and weak absorptions in the red/near-infrared, as expected for hyperporphyrin spectra. It should be noted that the *S* = 1/2 Compound I state is thought to involve an Fe(IV) center and an antiferromagnetically coupled radical that is delocalized over both the porphyrin and the thiolate axial ligand. The *S* = 1 Compound II state for this enzyme is believed to involve an Fe^IV^OH rather than Fe^IV^O center, which reflects the enhanced basicity of the latter group as a result of the thiolate ligand.

**Figure 8:**
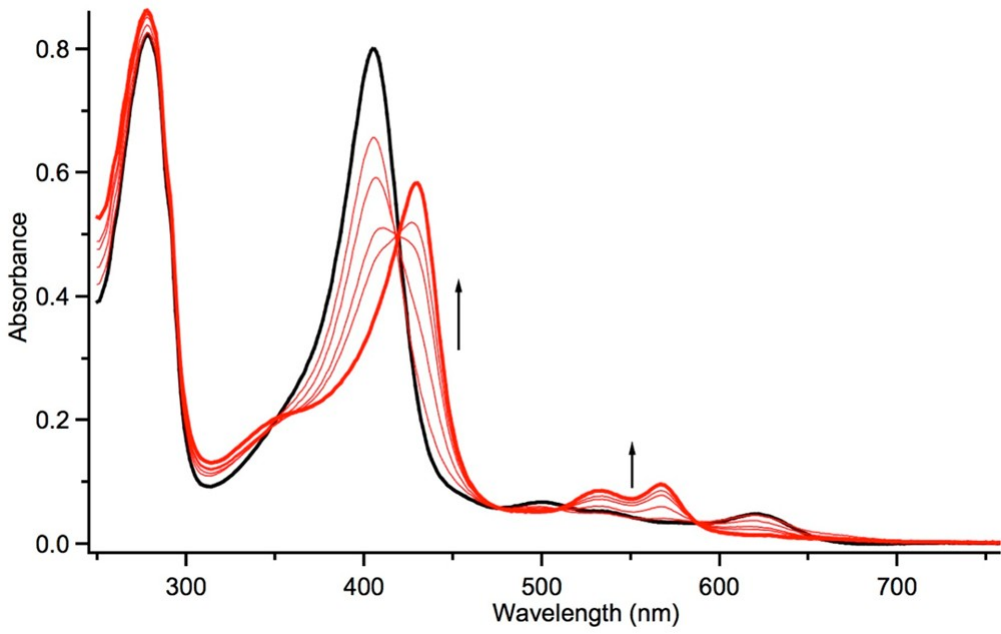
UV–vis spectra of HPC-II at pH 5 (50 mM citrate buffer, 500 mM NaCl) obtained from the reaction of the green ferric HPC enzyme and 12.5 equiv of peracetic acid. No significant buildup of Compound I occurred prior to Compound II formation. Adapted with permission from ref (32). Copyright 2016 American Chemical Society.

Figure 8 depicts the UV–vis spectrum of the Compound II intermediate of catalase (HPC-II), a tyrosinate-ligated heme enzyme from *Helicobacter pylori*.^32^ Unsurprisingly, the spectrum of this Fe^IV^OH species is qualitatively very similar to that of CPO-II (Figure 7b).

Importantly, the above spectra, and heme protein spectra in general, remain largely unassigned via modern quantum chemical calculations.

## 3 Protonated *meso*-Tetraarylporphyrins as Hyperporphyrins

**Figure 9:**
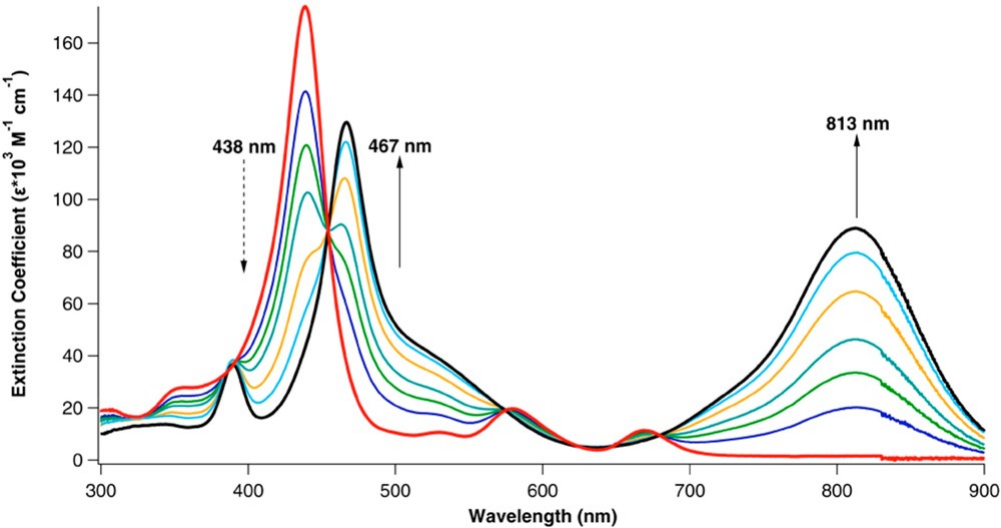
Acid titration of TAPP to the diprotonated state. Reproduced from ref (35). Copyright 2014 American Chemical Society.

Although Gouterman initially described only certain metalloporphyrins (and some metalloid derivatives) as hyperporphyrins, he and his co-workers later also included certain free-base derivatives as hyperporphyrins.^33^ A classic example involves the diprotonation of free-base 5,10,15,20-tetrakis(4-aminophenyl)porphyrin, H_2_[TAPP]. The diprotonated or “diacid” form, {H_4_[TAPP]}^2+^, exhibits significant bathochromic shifts of both the Q and B bands, as well as an extreme increase in intensity of the far-red Q band (Figure 9).^34,35^ In these cases, the charge-transfer effects obviously cannot be described as MLCT or LMCT but are entirely within the organic ligand; these will be called LLCT transitions.

Electronic communication between *meso*-aryl groups and the porphyrin ring is constrained by their relative spatial orientation. In simple free-base and metal-complexed TPP derivatives, steric interactions between the pyrrole β-hydrogens and the aryl *ortho*-hydrogens lead to significant twisting of the aryl groups out of the main porphyrin plane.^36^ Thus, aryl substituents typically exert only a modest influence on the electronic character of the porphyrin ring. In general, substituents exert comparable effects on the first oxidation and first reduction potentials, which leads to relatively constant electrochemical HOMO–LUMO gaps (defined as the algebraic difference between the oxidation and reduction potentials) and parallel Hammett plots.^37−39^ For a wide range of substituents, the absorption spectra of neutral tetra(*p*-X-phenyl)porphyrins (TXPPs) are also relatively consistent.^40^ With strongly electron-donating substituents (such as alkoxy and amino), however, there is a sharp break in the Hammett plots for oxidation potentials^39^ as well as a bathochromic shift of the Q bands,^40,41^ which can be interpreted as a gradual impingement of aryl-based MOs into the energy range of the porphyrin’s four Gouterman-type frontier MOs. Table 1 presents highlights of substituent effect data on the free-base porphyrins as well as for the diprotonated forms in which strong hyperporphyrin effects appear.

**Table 1:** Lowest-Energy Q Band Maxima (and Substituent-Induced Shifts) for Tetra(*p*-X-phenyl)porphyrins and Their Diprotonated Forms,^40^ Compared with Redox Potentials (and Substiuent-Induced Shifts) for the Tetra(*p*-X-phenyl)porphyrins,^39^ All in DMSO Solvent

		Q(0,0) λ_max_ (Δλ_max_, nm)		Redox Potentials (V vs SCE)	
*para* substituent X	Hammett σ_p_	H_2_[TXPP]	{H_4_[TXPP]}^2+^	*E*_ox_ (Δ*E*_ox_)	*E*_red_ (Δ*E*_red_)
–COOCH_3_	0.47	644 (−2)	656 (−3)	+1.14 (+0.10)	–0.92 (+0.11)
–H	0.00	646 (0)	659 (0)	+1.04 (0)	–1.03 (0)
–OCH_3_	–0.28	651 (+5)	696 (+37)	+0.94 (−0.10)	–1.08 (−0.05)
–NH_2_	–0.57	669 (+23)	811 (+152)	+0.48 (−0.56)	–1.18 (−0.15)

The case of TAPP (the -NH_2_ data in Table 1) illustrates that even neutral H_2_[TAPP] may be viewed as an incipient hyperporphyrin, with significant shifts of its Q band and oxidation potential (which serves as an indicator of the relative energy position of the HOMO). Yet, the magnitude of the hyperporphyrin effect induced by protonation is dramatically larger (Figure 9). Diprotonation of the porphyrin ring induces strong nonplanarity (saddling) of the porphyrin ring, primarily as a result of steric repulsion among the four internal pyrrole hydrogens.^42,43^ Characteristic hyperporphyrin effects have been observed for a wide range of porphyrin diacids, including those derived from octaethylporphyrin and β-octahalogeno-*meso*-tetraarylporphyrins.^44^ The most distinctive cases of free-base hyperporphyrin spectra occur with TPP diacids with strongly electron-donating *para*-substituents, exemplified by the aforementioned {H_4_[TAPP]}^2+^ dication or its dimethylamino analogue. In these cases, protonation occurs preferentially on the central nitrogens even though the peripheral amino substituents are also basic.

### 3.1 Charge Transfer from Multiple Strong Electron Donors to Protonated Porphyrins (Aminophenylporphyrins)

**Chart 2:**
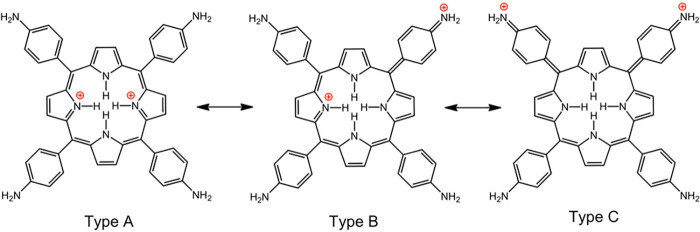
Resonance Forms Illustrating Charge Transfer from Aminophenyl to Protonated Porphyrin

Protonation of various *para*-aminophenylporphyrins has been the most thoroughly studied, with either amino^34,35^ or dimethylamino.^33^ as *para* substituents. Resonance forms for the doubly protonated porphyrin underscore transfer of electronic charge from the amino groups to the porphyrin ring; Chart 2 illustrates representative resonance forms of the three distinct types. We use the categorization proposed by Gouterman and co-workers when they first documented this type of hyperporphyrin.^33^ Type A depicts positive charges localized on the porphyrin pyrrole nitrogens; Type B shows one charge delocalized to one of the *para*-amino groups; and Type C shows both charges delocalized to two *para*-amino groups, which are necessarily *cis*. Types B and C have multiple different forms depending on which combinations of substituents are utilized.

Examination of the resonance forms offers a number of insights that correlate well with experimental observations.

(i) The aryl-porphyrin interactions can sustain up to two charge-transfer interactions of the type described, and *cis* rather than *trans* is required to delocalize the two charges. The magnitude of the hyperporphyrin effect can be most readily tracked by the position and extinction coefficient of the far-red Q band. With just two *para*-amino groups (the others being *para*-carbomethoxy), the *cis* regioisomer exhibits a hyperporphyrin band with λ_max_ at 763 nm (ε = 60 mM^–1^ cm^–1^), while for the *trans* regioisomer it is at 756 nm (ε = 41 mM^–1^ cm^–1^).^34^
(ii) The hyperporphyrin effect increases with additional donor substituents, where the tri- and tetrasubstituted cases offer multiple modes for the resonance delocalizations of both Type B and Type C. In the limit of four *para*-amino groups, the hyperporphyrin Q band appears at 813 nm (ε = 89 mM^–1^ cm^–1^) (Figure 9).^35,40^
(iii) Although monoprotonated porphyrins are rarely observed,^45^ they have been proposed for monoamino TPPs where the other three substituents are electron-withdrawing carbomethoxy^34^ or sulfonato groups.^46^ In these cases, Type B resonance forms are stabilized, but Type C are not, so the second protonation is retarded.
(iv) Excess acid ultimately protonates all the peripheral amino substituents and destroys the hyperporphyrin effect.^33,40^

Although the resonance forms are suggestive, the shapes and relative energies of the frontier MOs afford more detailed insight into the origin of the hyperporphyrin effect. The effect has been thought to arise for a single *para*-aminophenyl group via elevation of an aminophenyl-based MO to the level of molecular HOMO; in the tetrasubstituted case, both the HOMO and HOMO–1 have been thought to be aminophenyl-based.^47^ Thus, the hyperporphyrin transition is described as an aminophenyl-to-porphyrin charge transfer, as suggested by the resonance forms. This picture holds up moderately well, but far from perfectly, in light of modern DFT calculations. NMR,^48,49^ FTIR, and resonance Raman^44,50^ studies of hyperporphyrin systems support the key structural features implied by the resonance forms, i.e., notably enhanced bonding between the porphyrin *meso* carbon and the aryl *ipso* carbon.

### 3.2 Origins of the Hyperporphyrin Effect: Recent TDDFT Results

**Chart 3:**
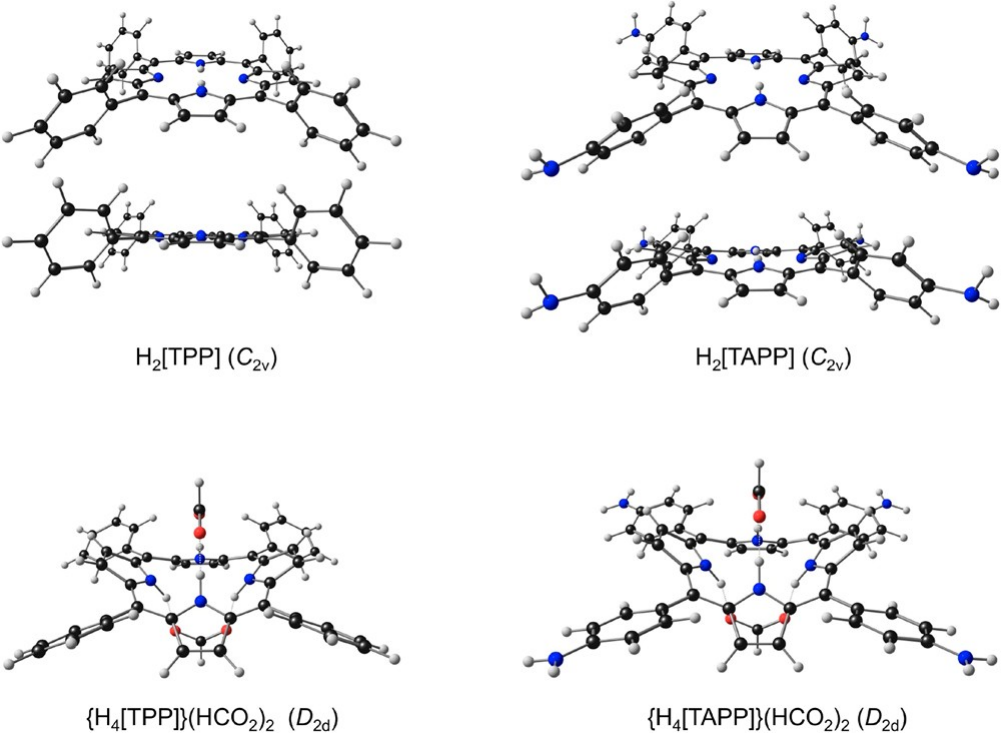
Ball-and-Stick Representations of the Optimized Geometries of TPP and TAPP Derivatives Studied in a Recent DFT/TDDFT Study, Reproduced from Ref (51); Copyright 2021 American Chemical Society

**Figure 10:**
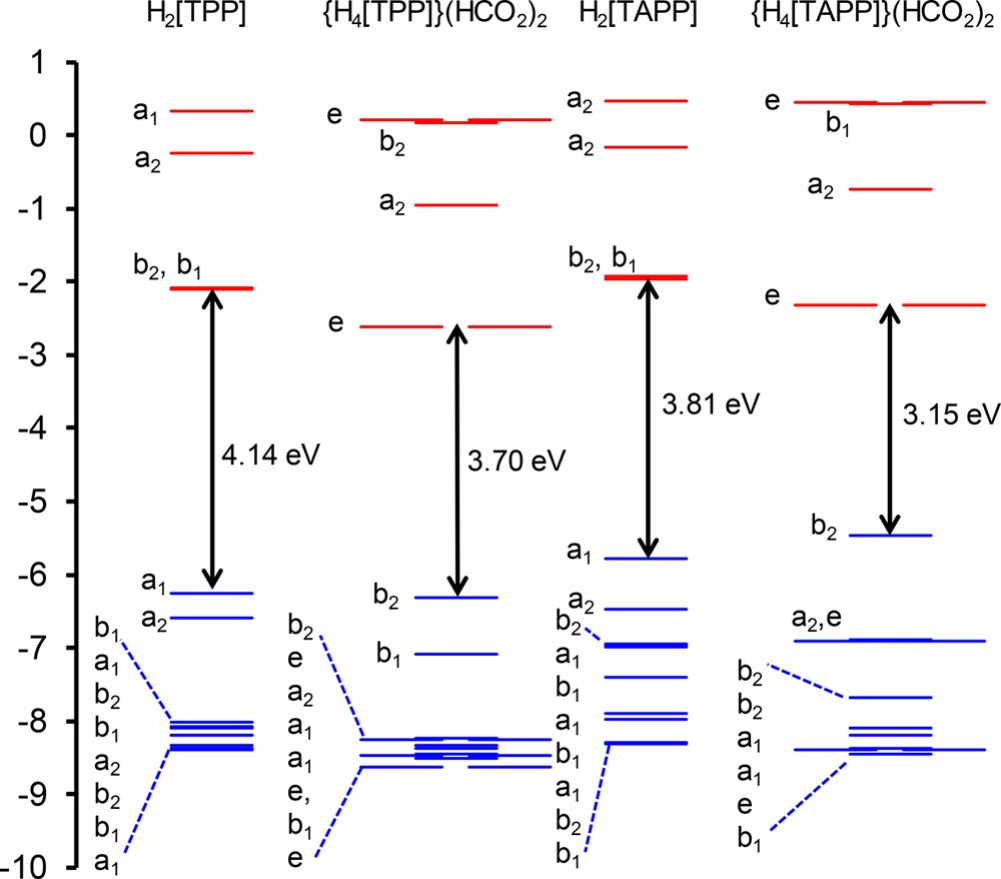
CAMY-B3LYP/STO-TZ2P Kohn–Sham MO energy (eV) level diagram for the four species studied, with the solvent (dichloromethane) modeled with COSMO. The irreps refer to the point groups indicated in Chart 3. Briefly, the *D*_4h_ irreps a_2u_ and a_1u_ transform as a_1_ and a_2_, respectively, for the *C*_2v_ point group used for the free-base porphyrins, and as b_2_ and b_1_, respectively, for the *D*_2d_ point group of the diacids. Reproduced from ref (51). Copyright 2021 American Chemical Society.

**Figure 11:**
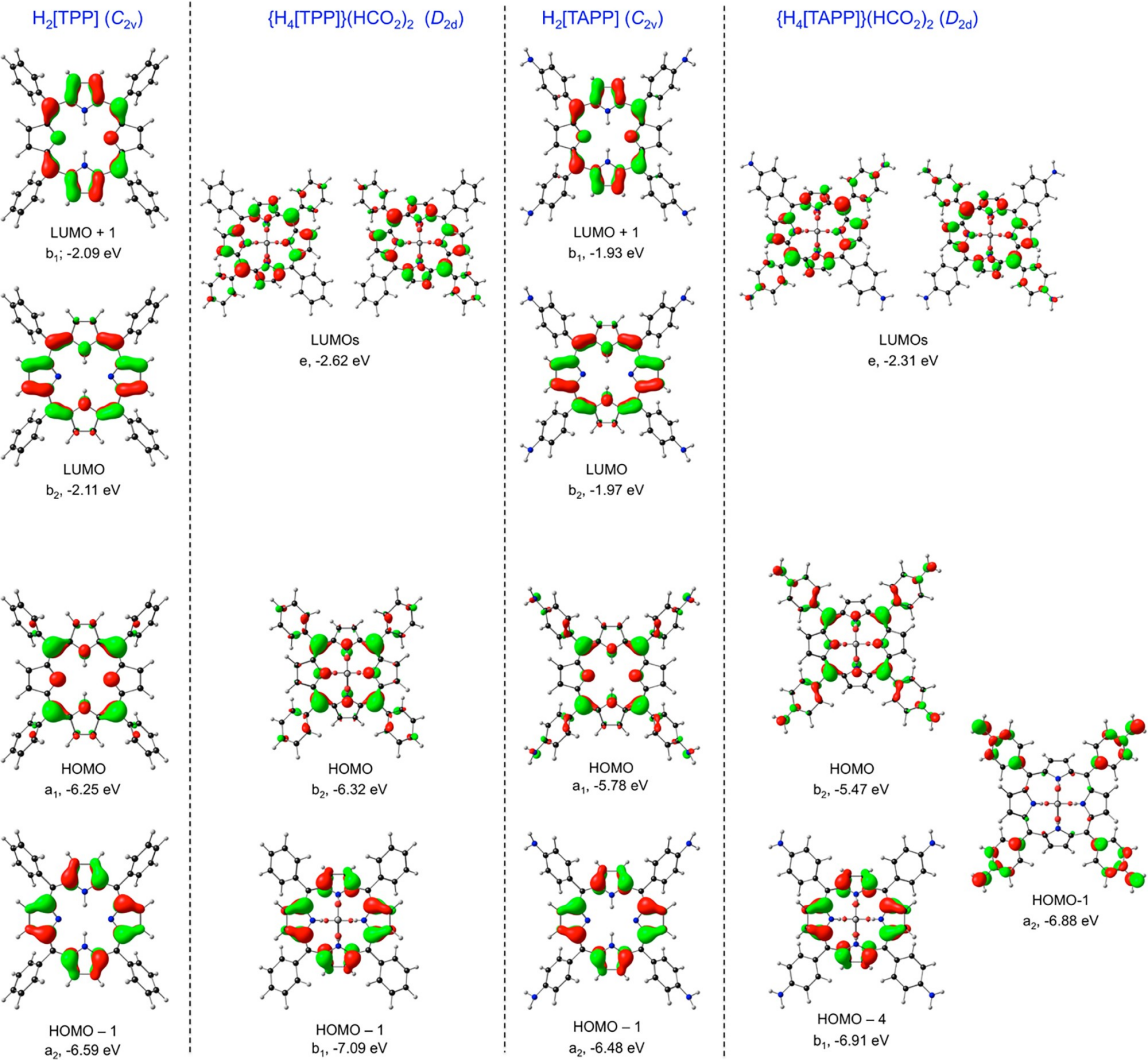
Selected CAMY-B3LYP (COSMO) frontier MOs, along with their irreps and orbital energies, relevant to Figure 10. Reproduced from ref (51). Copyright 2021 American Chemical Society.

We have recently reported a TDDFT study aimed at a better understanding of the dramatic hyperporphyrin spectrum of TAPP diacid.^51^ Toward this end, we studied both TPP and TAPP (both symmetrized to *C*_2v_) and their diacids, the latter as their highly symmetric (*D*_2d_) bisformate complexes (Chart 3). We found it essential to employ both a hybrid functional (such as B3LYP or CAMY-B3LYP) and a solvation scheme (in this case COSMO with CH_2_Cl_2_) to obtain good simulations of experimental spectra. The results, highlighted here by relevant MO energy level diagrams (Figure 10) and plots of the relevant MOs (Figure 11), led to several concrete insights, including multiple factors manifesting themselves as hyperporphyrin spectra.

For all four species studied, both free bases and diacids, the Q band consists primarily of HOMO(a_2u_)-to-LUMO/LUMO+1 transitions. Two different effects appear to account for the Q band redshifts. For diprotonation of the free-base forms, the major factor underlying Q band redshifts is a lowering of the LUMOs as a result of infusion of *meso*-aryl character. Elevation of the “a_2u_” HOMO plays a smaller role. In contrast, the redshifted Q band of free-base H_2_[TAPP] relative to H_2_[TPP] reflects destabilization of the “a_2u_” HOMO because of antibonding “filled–filled” interactions with aminophenyl-based occupied MOs, while the LUMOs are less affected energetically.

Beyond the Q bands (i.e., for the Soret bands as well as certain pre-Soret and post-Soret bands), the transitions of the diacid forms are more complex, with *meso*-aryl → LUMO character mixing in with classic Gouterman “a_1u_” → LUMO transitions. Indeed, some of these transitions may be described as primarily *meso*-aryl or aminophenyl-based.

### 3.3 Charge Transfer from Deprotonated Electron Donors to Neutral Porphyrins (Hydroxyphenylporphyrins)

**Chart 4:**
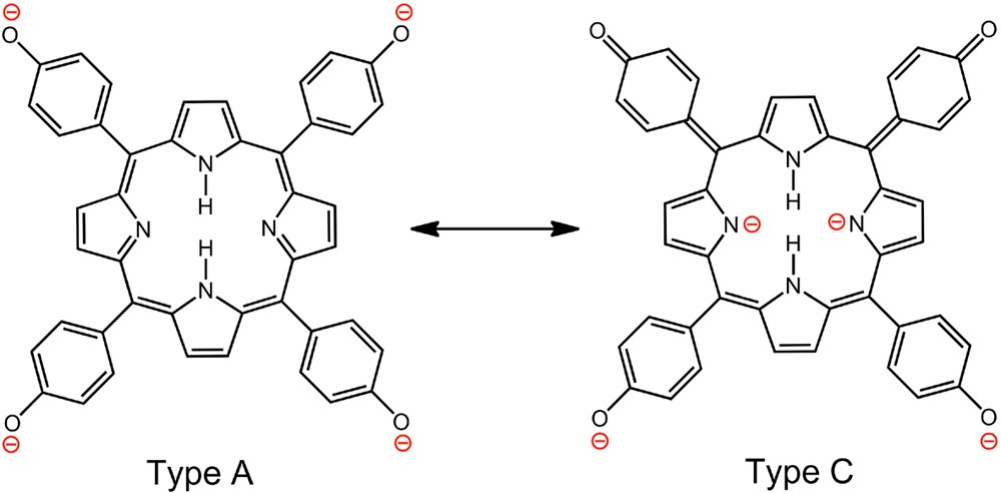
Representative Resonance Forms Illustrating Charge Transfer from Phenoxide to Neutral Porphyrin

5,10,15,20-Tetrakis(*p*-hydroxyphenyl)porphyrin (THPP) and related derivatives have been studied extensively by Milgrom and others for many years because of their unusual property of being very easily oxidized to phenoxy radicals, even in air for some derivatives.^52^ This observation already suggests that the HOMO is localized on the hydroxyphenyl group and that spectrophotometric titrations of H_2_[THPP] and analogues must be carried out with the careful exclusion of oxygen. Base titrations of TPPs with one to four *para*-hydroxy groups show clear hyperporphyrin spectra,^53^ assigning the dianionic forms to hyperporphyrins via the resonance structures shown in Chart 4. Resonance forms of Type B are not shown but are also possible. In this case, an anionic, strongly electron-donating group is generated via the deprotonation of the phenol substituents while the porphyrin remains uncharged. Similar hyperporphyrin effects have also been demonstrated with Ni[THPP] in a strong base.^44^

In the case of monosubstituted *p*-hydroxyphenylporphyrin, the choice of solvent can affect whether a hyperporphyrin spectrum is observed. In DMF, deprotonation leads to the expected hyperporphyrin spectrum, but in 50% aqueous DMF, there is no hyperporphyrin effect, presumably as a result of strong hydrogen bonding that lowers the orbitals of the phenoxide group so they are no longer the HOMO.^50^ Analogous to the case of excess acid with protonated hyperporphyrins, excess base can deprotonate the porphyrin core of H_2_[THPP] and thereby destroy the hyperporphyrin effect.^54^

### 3.4 Push–Pull Charge Transfer through Protonated Porphyrins (Aminophenyl/Pyridylporphyrins)

Type C resonance forms (Charts 3 and 4) feature two double bonds that are exocyclic to the porphyrin ring. In the limit of four such exocyclic double bonds, the derivatives are called oxoporphyrinogens, which are generally formed via the oxidation of porphyrins.^55^ The most common example of an oxoporphyrinogen is that formed by oxidation of 5,10,15,20-tetrakis(3,5-*t*-butyl-4-hydroxyphenyl)porphyrin, a THPP analogue that incorporates the steric hindrance of *t*-butyl groups to stabilize the oxidized form. Because the pyrrole nitrogens in these cases are readily derivatized, these oxoporphyrinogens have been studied as catalysts and sensors, as well as in other applications.^56^

**Figure 12:**
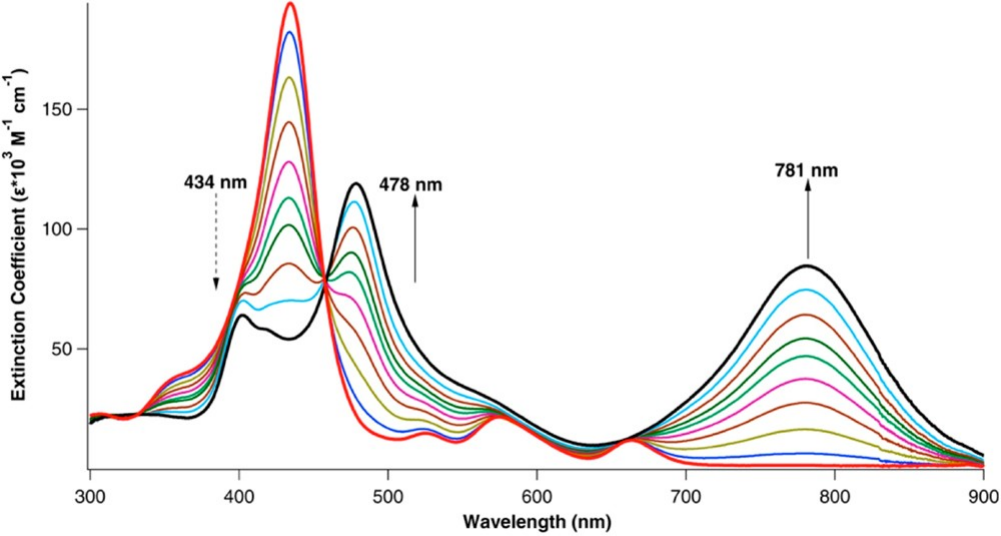
Acid titration of 5,10,15-tris(4-aminophenyl)-20-pyridylporphyrin, H_2_[TA_3_PyP]. The hyperporphyrin spectrum corresponds to the triprotonated state, {H_5_[TA_3_PyP]}^3+^. Reproduced from ref (35). Copyright 2014 American Chemical Society.

One case of a hyperporphyrin with a proposed oxoporphyrinogen resonance form has been reported, in which the additional exocyclic double bonds are formed by charge transfer through the porphyrin core between an electron-rich aryl group and an electron-deficient aryl group. Thus, acid titration of 5,10,15-tris(4-aminophenyl)-20-pyridylporphyrin (H_2_[TA_3_PyP]) shows a hyperporphyrin spectrum at the triprotonated state (Figure 12).^35^

**Chart 5:**
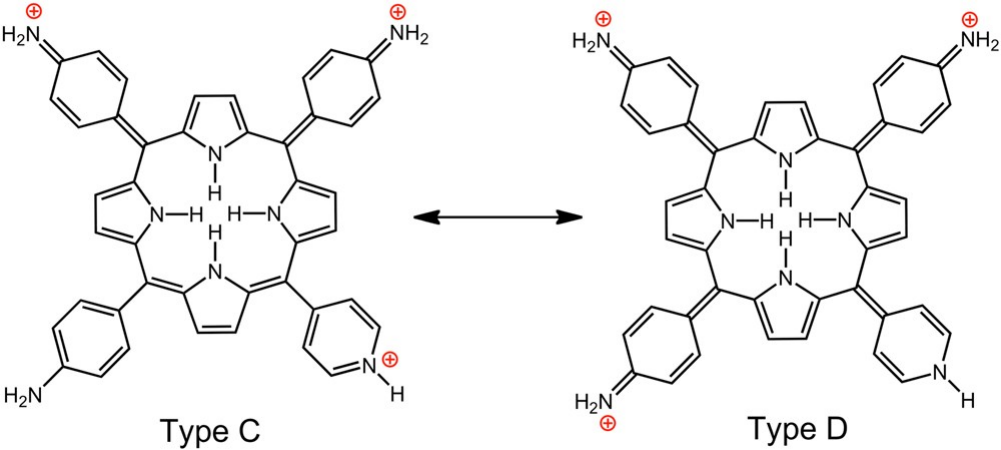
Resonance Forms Illustrating Aryl–Aryl Charge Transfer through the Porphyrin Core

The diprotonated state is not observed during the titration, as indicated by clean isosbestic points during the titration from +1 to the +3 state. At the triprotonated stage, two aminophenyl groups delocalize the positive charge from the two interior protonations, while the third protonation forms a pyridinium group that can interact with the third aminophenyl group in a push–pull charge transfer across the porphyrin ring (Chart 5). This resonance form with four exocyclic double bonds represents a new type for hyperporphyrins (Type D).

This novel type of hyperporphyrin effect is quite strong; the Q band appears at 781 nm (ε = 84 mM^–1^ cm^–1^),^35^ with a λ_max_ comparable to that of a triamino-TPP at 784 nm (ε = 53 mM^–1^ cm^–1^),^34^ but an intensity comparable to that of TAPP at 813 nm (ε = 89 mM^–1^ cm^–1^).^35^

### 3.5 Enhanced Charge Transfer via Ethynyl Linkers (Arylethynylporphyrins)

Aryl-to-porphyrin charge transfer interactions are enhanced when ethynyl linkers are inserted between the porphyrin *meso* positions and phenyl substituents, allowing for greater coplanarity of the two rings. The cases studied included *para*-dimethylamino^57^ and *para*-hydroxy substituents,^58^ but were limited by the synthetic method to two ethynyl-linked aryl groups located *trans* to one another. For protonation of the porphyrin with *trans* dimethylaminophenyls and ethynyl linkers, the Q band at 802 nm (ε = 49 mM^–1^ cm^–1^) was found to be considerably more redshifted (but less intense) than that in the analogue without the ethynyl linkers (723 nm, ε = 60 mM^–1^ cm^–1^). The corresponding porphyrins with *para*-hydroxyphenyl groups were studied in both acidic and basic media. In acidic medium, the hyperporphyrin effect of hydroxy groups was found to be smaller but nonetheless enhanced with the ethynyl linkers (736 nm with ethynyl linkers and 671 nm without). In basic medium, the λ_max_ values are 752 nm with ethynyl linkers and 671 nm without.

### 3.6 Hyperporphyrins Based on Charge Transfer from Porphyrin to Aryl Have Not Been Observed

**Chart 6:**
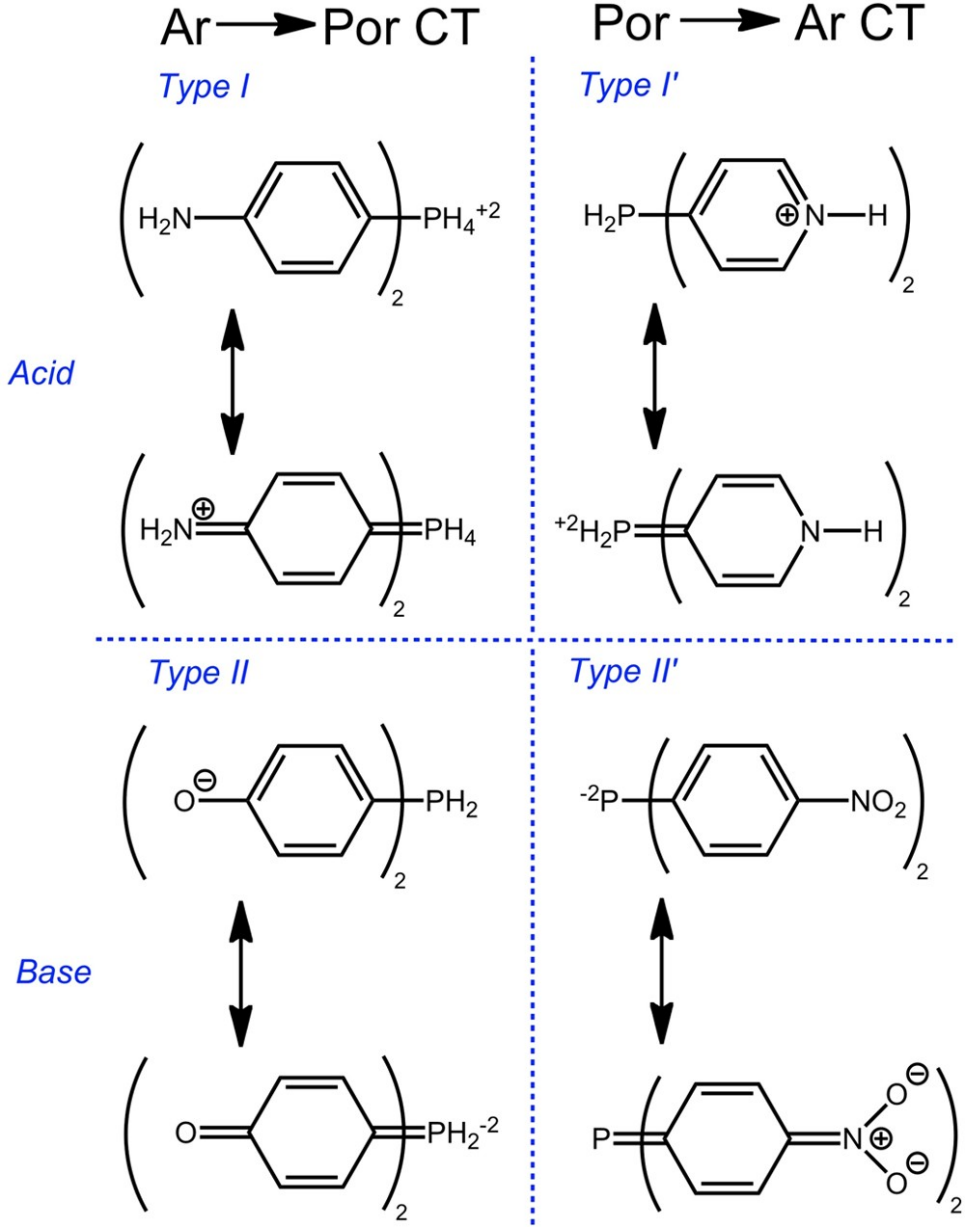
Hyperporphyrins from *meso*-Tetraarylporphyrins via Acid/Base Reactions

In theory, one can imagine a variety of different circumstances in which charge transfer can be induced between a porphyrin and an attached aryl group, as illustrated in Chart 6.

In this paper, we have cited both types of aryl-to-porphyrin LLCT, specifically protonated TAPP as emblematic of Type I and deprotonated THPP of Type II. To our knowledge, hyperporphyrins in which charge transfer goes from porphyrin to aryl substituents have not yet been reported in the literature. Protonation of pyridylporphyrins (and *N*-methylpyridiniumyl) do not show hyperporphyrin effects as suggested in Type I′. Deprotonation should make the porphyrin ring a much stronger electron donor as suggested in Type II′. However, deprotonation of tetrakis(4-nitrophenyl)porphyrin (TNPP) was reportedly unsuccessful using TBAOH in various solvents.^59^ Tetrakis(*N*-methyl-4-pyridiniumyl)porphyrin (TMPyP) has been reported to be deprotonated in aqueous solution with an apparent p*K*_a_ of 12.6; however, the proposed result was a single deprotonation and an unremarkable spectrum.^60−62^ The interior pyrrolic hydrogens of porphyrins are weakly acidic, even with strong electron-withdrawing substituents, and it may be that stronger base systems will be required to observe a hyperporphyrin with a porphyrin-to-aryl charge-transfer.

Beyond the four types of LLCT interactions described in Chart 6, one could imagine a push–pull hyperporphyrin that did not rely upon either protonation or deprotonation. In such a case, the presence of both strong electron donation and electron withdrawal at different sites on a porphyrin ring could lead to a push–pull resonance form analogous to that shown as Type D in Chart 5. A large number of porphyrins, including some with appropriate push–pull substituents, have been prepared for different applications, for example, as anticancer agents, but none showed hyperporphyrin behavior in aqueous solution.^63^ A copper porphyrin with *trans meso* substituents of dimethylaminophenylethynyl and nitrophenylethynyl also showed no hyperporphyrin effects.^64^ We are not aware of any free-base porphyrins that show hyperporphyrin effects without the assistance of an acid or base.

### 3.7 Hyperporphyrins in Redox and Photoredox Reactions

**Chart 7:**
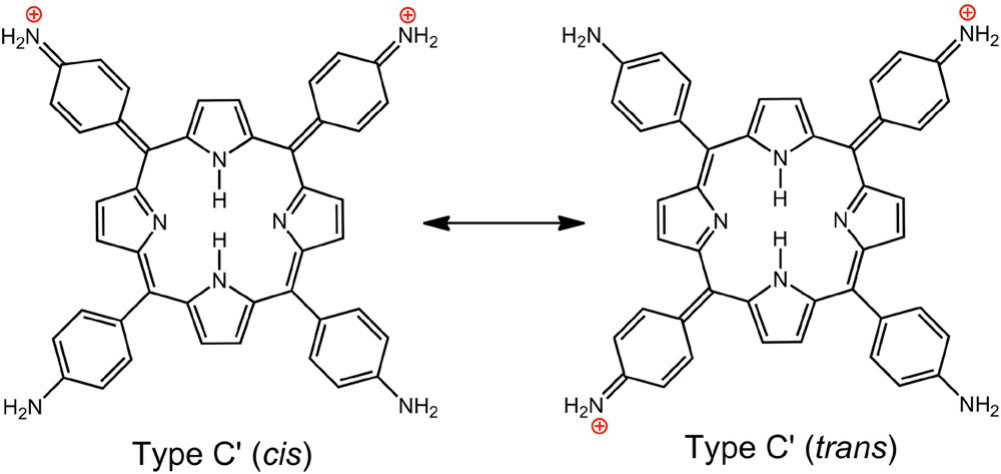
Resonance Forms after Two-Electron Oxidation of TAPP

Aside from protonation reactions, porphyrins can also acquire positive charges via oxidation. Here, again, strongly electron-donating groups engender hyperporphyrin effects. Hyperporphyrin effects in the oxidation of H_2_[TAPP] have been tracked by oxidative titration or by spectroelectrochemistry.^65,66^ For the doubly oxidized species {H_2_[TAPP]}^2+^, both *cis* and *trans* resonance forms of Type C′ are possible (Chart 7).

The oxidation of H_2_[TAPP] is complicated by polymerization, analogous to the oxidative polymerization of aniline. Reversible electrochemical oxidation of a poly-TAPP film also shows clear hyperporphyrin spectra at positive potentials. In this case, individual porphyrin units in the polymer can still be described as having characteristic exocyclic Type C′ resonance forms; in fact, these structures are considered critical to the electronic conductivity observed for poly-TAPP films.^65,66^

The polymerization of TAPP is minimized in acidic media, and titration of the monomeric, fully protonated species {H_8_[TAPP]}^6+^ with ammonium persulfate in aqueous acid results in typical hyperporphyrin spectra upon oxidation.^66^ In acidic media, protonated amino substituents would ordinarily fail to yield hyperporphyrin effects. In this case, however, oxidation followed by the loss of two protons generates a +6 form that can still sustain hyperporphyrin resonance, i.e., Type C′ forms in which additionally both pyrroles and both anilino groups are protonated.

### 3.8 Free-Base Hypercorroles

**Figure 13:**
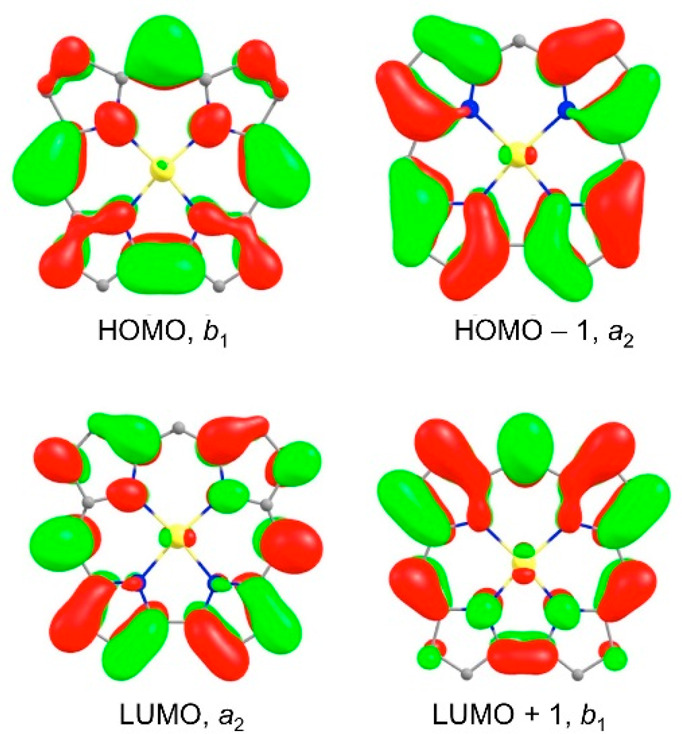
Gouterman frontier MOs of an unsubstituted Au corrole. Reproduced from ref (67). Copyright 2017 American Chemical Society.

The Gouterman four-orbital model has been successfully applied to corroles; the four frontier orbitals of unsubstituted gold corrole, Au[Cor], are depicted in Figure 13.^9,67^ The spectra of free-base corroles present a number of interpretational challenges. The molecules are not only strongly nonplanar as a result of steric repulsion among the three central hydrogens, but they also exist as a mixture of two tautomers. Furthermore, free-base corroles are partially to fully ionized in many common solvents (such as DMF and DMSO), even in the absence of an added base.^68−70^ Free-base corroles, however, can only undergo a single protonation at their cores, and the protonated site can only interact with three *meso* substituents. While these features distinguish free-base corroles from porphyrins, many of the same principles of hyperporphyrin spectra apply.

**Figure 14:**
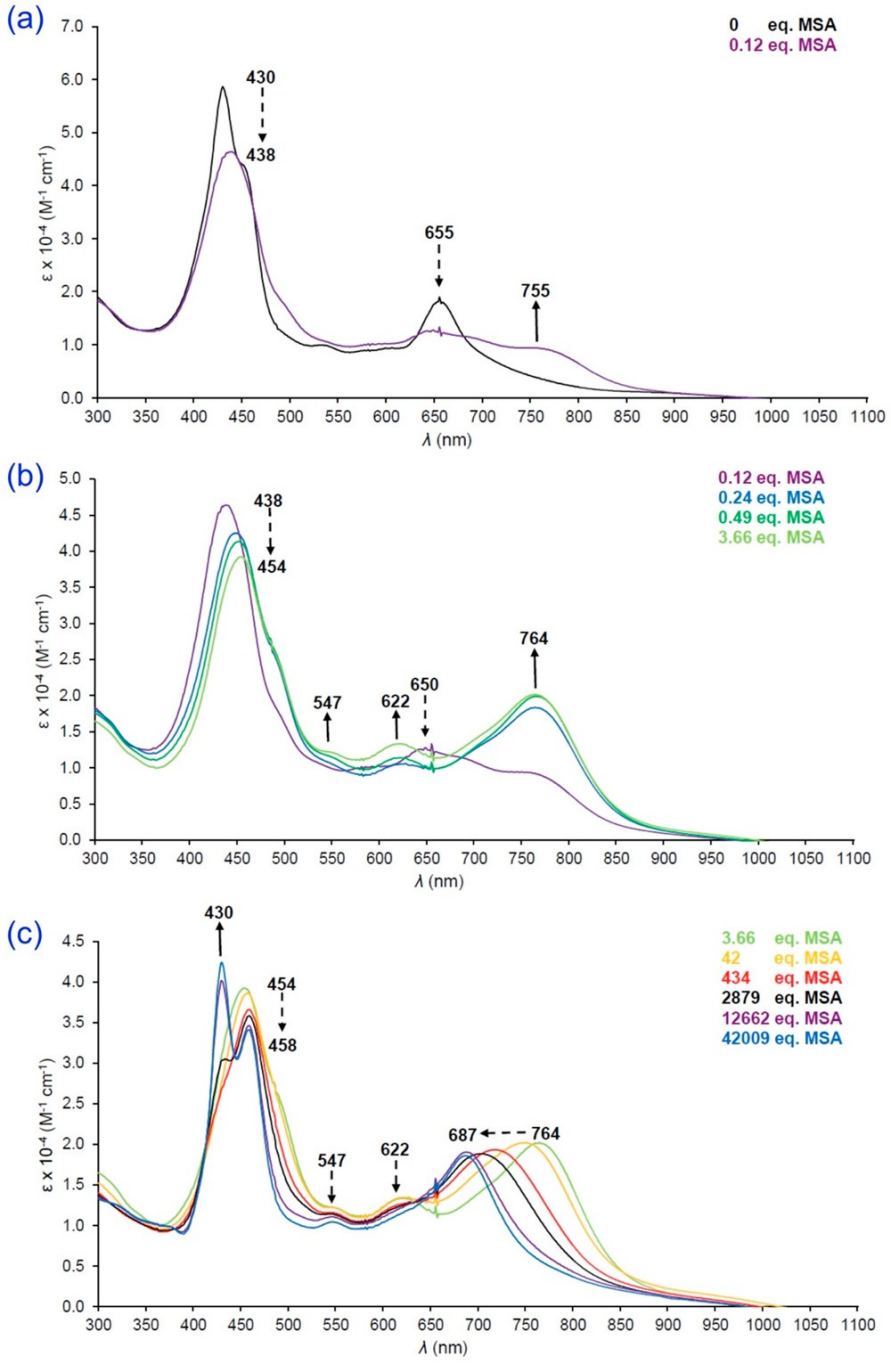
Spectral changes during the acid titration of H_3_[TAPC] (MSA = methanesulfonic acid). Reproduced from ref (71). Copyright 2021 American Chemical Society.

Acid titrations of all the isomers (*o,m,p*) of *meso*-tris(aminophenyl)corrole, H_3_[TAPC], have been studied.^71^ Comparison of the *para* TAPC isomer with the corresponding porphyrin (TAPP) is particularly instructive. Successive protonations (Figure 14) lead first to neutralization of the anion to form the neutral corrole and subsequently to the monoprotonated form {H_4_[TAPC]}^+^ with clear hyperporphyrin (hypercorrole) characteristics. Upon treatment with excess acid, the spectrum returns to normal.

**Chart 8:**
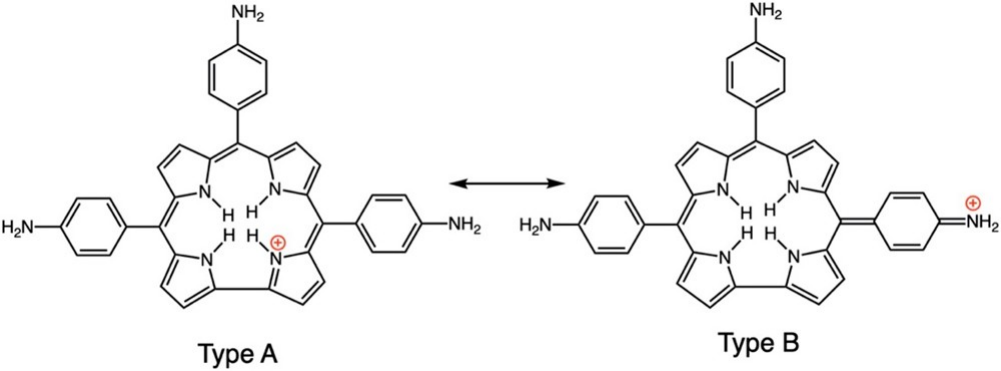
Resonance Forms for {H_4_[TAPC]}^+^

Chart 8 depicts the resonance forms of the *para* isomer of H_4_[TAPC]}^+^; one localizes the positive charge on a pyrrole nitrogen (Type A), and there are three options for delocalizing the charge to aminophenyl groups (Type B). Unlike for TAPP diacid (see Chart 2), a Type C resonance form is not possible. The resonance forms suggest that {H_4_[TAPC]}^+^ has fewer pathways for charge delocalization interactions (as well as fewer aminophenyl substituents) relative to TAPP diacid. The net result is that the hyperporphyrin effect in TAPP diacid (see Figure 9) is distinctly stronger than what is observed for {H_4_[TAPC]}^+^. Thus, the Q band positions and extinction coefficients are 813 nm (89 mM^–1^ cm^–1^) for TAPP diacid^35^ and 764 nm (20 mM^–1^ cm^–1^) for {H_4_[TAPC]}^+^.^71^ As the peripheral amino groups become successively protonated (Figure 14c), the effects gradually diminish, without clear isosbestic points since multiple species are present, and the spectrum ultimately returns to a normal Q band at 687 nm.

## 4 Metallotriarylcorroles

**Figure 15:**
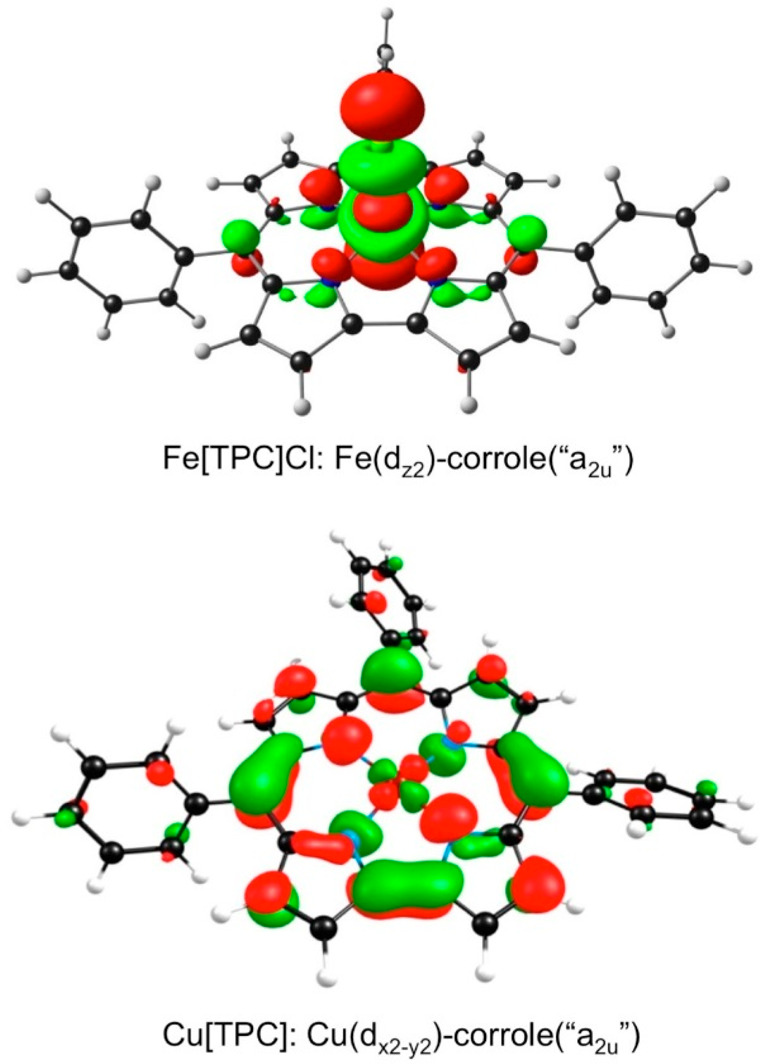
Two paradigmatic metal(d)-corrole(π) orbital interactions responsible for ligand noninnocence. Reproduced from ref (67). Copyright 2017 American Chemical Society.

While many 4d and 5d corroles presumably exhibit hypso spectra (a point that still needs verification),^10^ many first-row transition metal corroles clearly exhibit d-type hyper spectra.^24,72^ Among the latter, many (but not all) *meso*-triarylcorrole derivatives exhibit a remarkable substituent effect, which is not observed for *meso*-tetraarylporphyrins. The Soret maximum in these systems redshifts systematically with increasing electron-donating character of the *para* substituent on the *meso*-phenyl groups. Such substituent effects are particularly well-established for Mn, Fe, Co, and Cu corroles (Table 2). A variety of probes have established that the effect is specific to noninnocent metallocorroles, i.e., those having partial corrole^•2–^ radical character, which typically arises via one of two orbital interactions depicted in Figure 15.^67,72^ Although few of these spectra have been theoretically analyzed, a TDDFT study of copper triarylcorroles suggests that the substituent-sensitive components of the Soret manifolds are aryl-to-corrole^•2–^ charge transfer transitions,^73^ not unlike a number of LLCT transitions mentioned above. Some of the main classes of noninnocent metallocorroles are described below.

**Table 2:** Soret Maxima (nm) of Different *meso*-Triarylcorrole (TArC) Derivatives, Including *meso*-Tris(*para*-X-phenyl)corrole (X = OMe, Me, H, and CF_3_) Derivatives, Adapted from Ref (67); Copyright 2017 American Chemical Society.^a^

M[TArC](L)_n_	Ar = *p*OMeP	Ar = *p*MeP	Ar = Ph (TPC)	Ar = *p*CF_3_P	Ar = C_6_F_5_
*Noninnocent metallocorroles*					
Cu[TArC]	433	418	413	407	406
Cu[F_8_TArC]	436	421	409	401	
Cu[Br_8_TArC]	468	453	439	436	442
Cu[(CF_3_)_8_TArC]	507	471	459		
Mn[TArC]Cl	460	442	433	423	414
Fe[TArC]Cl	426	419	410	401	370, 396
Fe[F_8_TArC]Cl	367	360	355	353	
Fe[TArC](NO)	416	400	390	385	378
Fe[Br_8_TArC](NO)	394	395	397	391	392
{Fe[TArC]}_2_O	375, 410	389	386	383	382
Co[TArC](PPh_3_)	399	392	387	385	376, 408
Co[Br_8_TArC](PPh_3_)	423	418	412	421	
Co[TArC](py)	402	393	388	386	
Co[Br_8_TArC](py)	392	391	392	396	
Ag[Br_8_TArC]	450	438	425	416, 448	
Pt[TArC](Ar^1^)(Ar^2^)	475	460	453	443	
*Innocent metallocorroles*					
Cr[TArC](O)	404	404	403	404	
Mn[TArC]Ph	387	389	394	398	
Fe[TArC]Ph	385	383	383	384	
Co[TArC](py)_2_	434, 453	437, 453	437, 452	442, 453(sh)	440
Co[Br_8_TArC](py)_2_	446, 462	445, 461	445, 461	447, 460	
Mo[TArC](O)	440	439	438	439	
Mo[TArC]_2_	350	362	356		
Rh[TArC](PPh_3_)	427	430	429	431	428
Ag[TArC]	423	423	423	423	421
W[TArC]_2_		359	357	356	
Ru[TArC]NO	404	404	404	404	
Ru[TArC]N	419	418	418	417	
{Ru[TArC]}_2_	329, 406	329, 398	328, 397	328, 397	
Re[TArC](O)	441	440	439	438	
Tc[TArC](O)	413	412	410	410	
Os[TArC](N)	445	443	442	441	
{Os[TArC]}_2_	286, 407	287, 407	287, 405	287, 407	
Pt[TArC](Ar^1^)(PhCN)	427	427	426	430	
Pt[TArC](Ar^1^)(py)		430	427, 437	427, 438	
Au[TArC]	420	420	418	419	421
Au[Br_8_TArC]	431	430	429	429	428

a Ar, Ar^1^, and Ar^2^ refer to different aryl groups.

### 4.1 The Manganese Case

**Figure 16:**
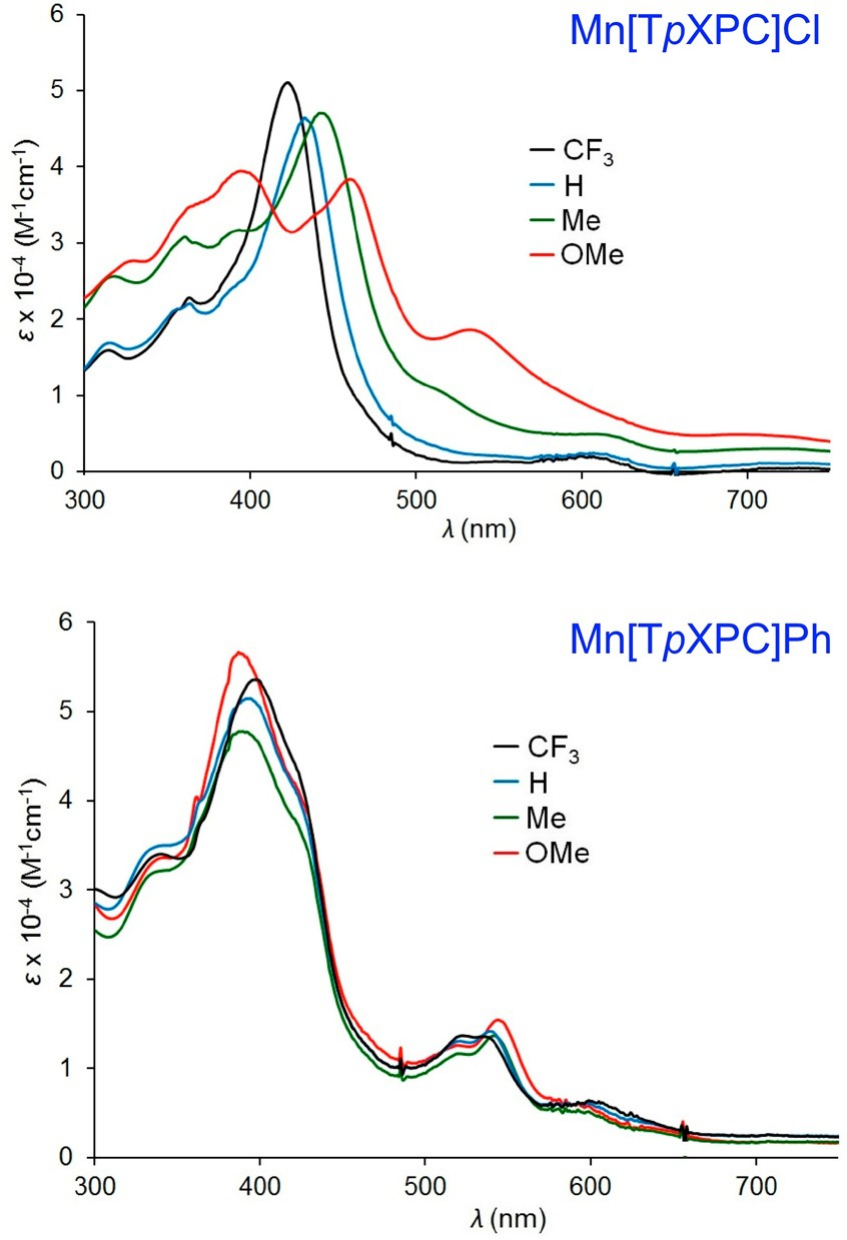
Electronic absorption spectra (in dichloromethane) of Mn[T*p*XPC]Cl and Mn[T*p*XPC]Ph derivatives. Reproduced from ref (74). Copyright 2018 American Chemical Society.

While all Mn corroles exhibit complex d-type hyper spectra relative to “normal” Al and Ga corroles, the Mn[T*p*XPC]Cl series exhibits substituent-sensitive Soret bands, implicating a noninnocent Mn^III^-corrole^•2–^ description. In contrast, the Mn(III) and Mn[T*p*XPC]Ph series (Figure 16) are thought to involve an innocent corrole.^74^

### 4.2 The Iron Case

**Figure 17:**
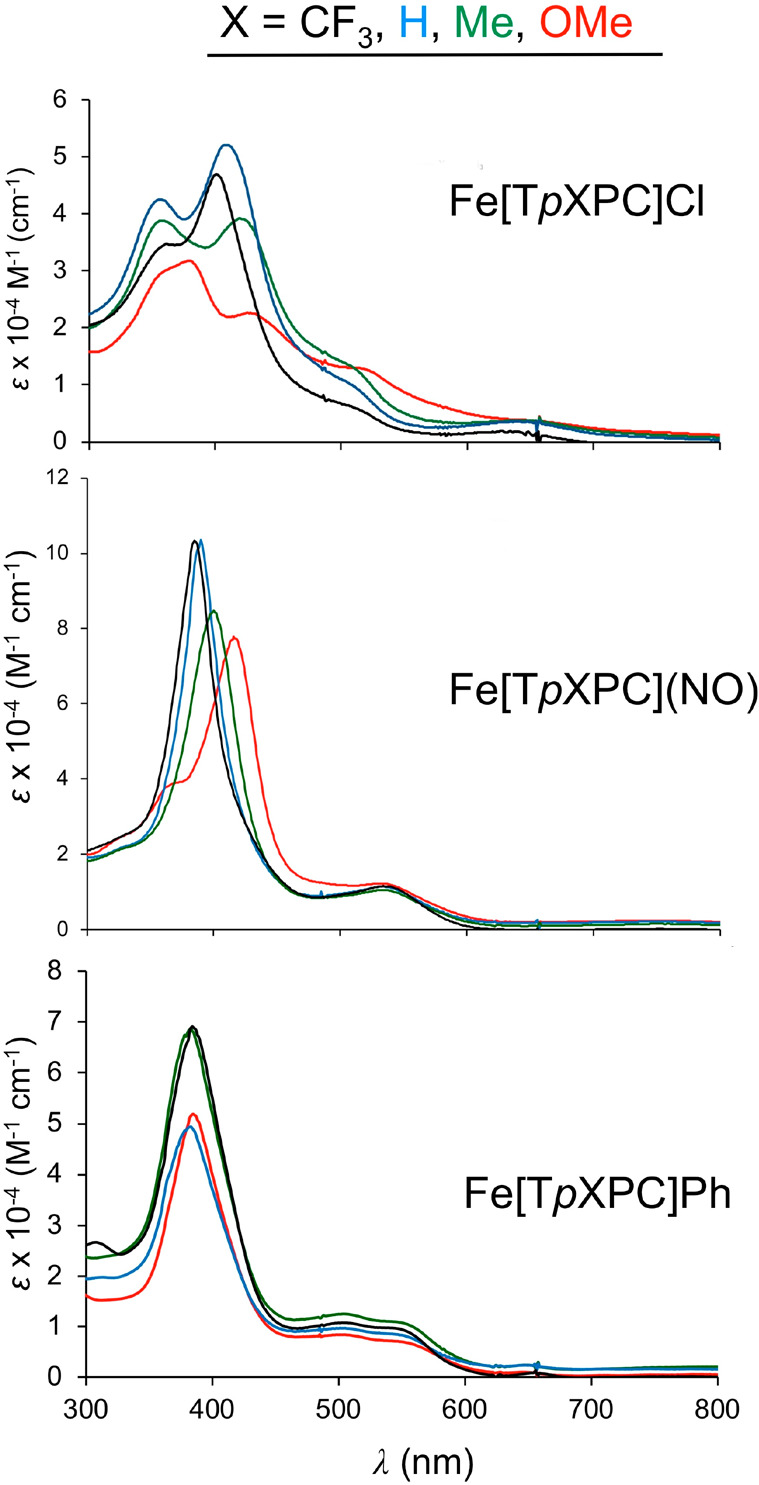
UV–vis spectra of three series of iron *meso*-tris(*para*-X-phenyl)corrole, Fe[T*p*XPC](L), where L = Cl, NO, and Ph. Reproduced from ref (72). Copyright 2019 American Chemical Society.

In an exact parallel to Mn triarylcorroles, FeCl^75−77^ triarylcorroles exhibit substituent-sensitive Soret maxima clearly indicative of aryl-to-corrole LLCT transitions and hyperporphyrin character, but FePh triarylcorroles do not (Figure 17). In an unexpected development, FeNO^78^ triarylcorroles were found to exhibit substituent-sensitive Soret maxima, suggesting a novel {FeNO}^7^-corrole^•2–^ description, which was later supported by several other lines of evidence. Similarly, μ-oxo diiron triarylcorroles also exhibit mildly substituent-sensitive Soret maxima, suggesting the following intramolecularly spin-coupled description
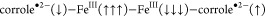
which was also supported by broken-symmetry DFT calculations.^79^

### 4.3 Cobalt Corroles

**Figure 18:**
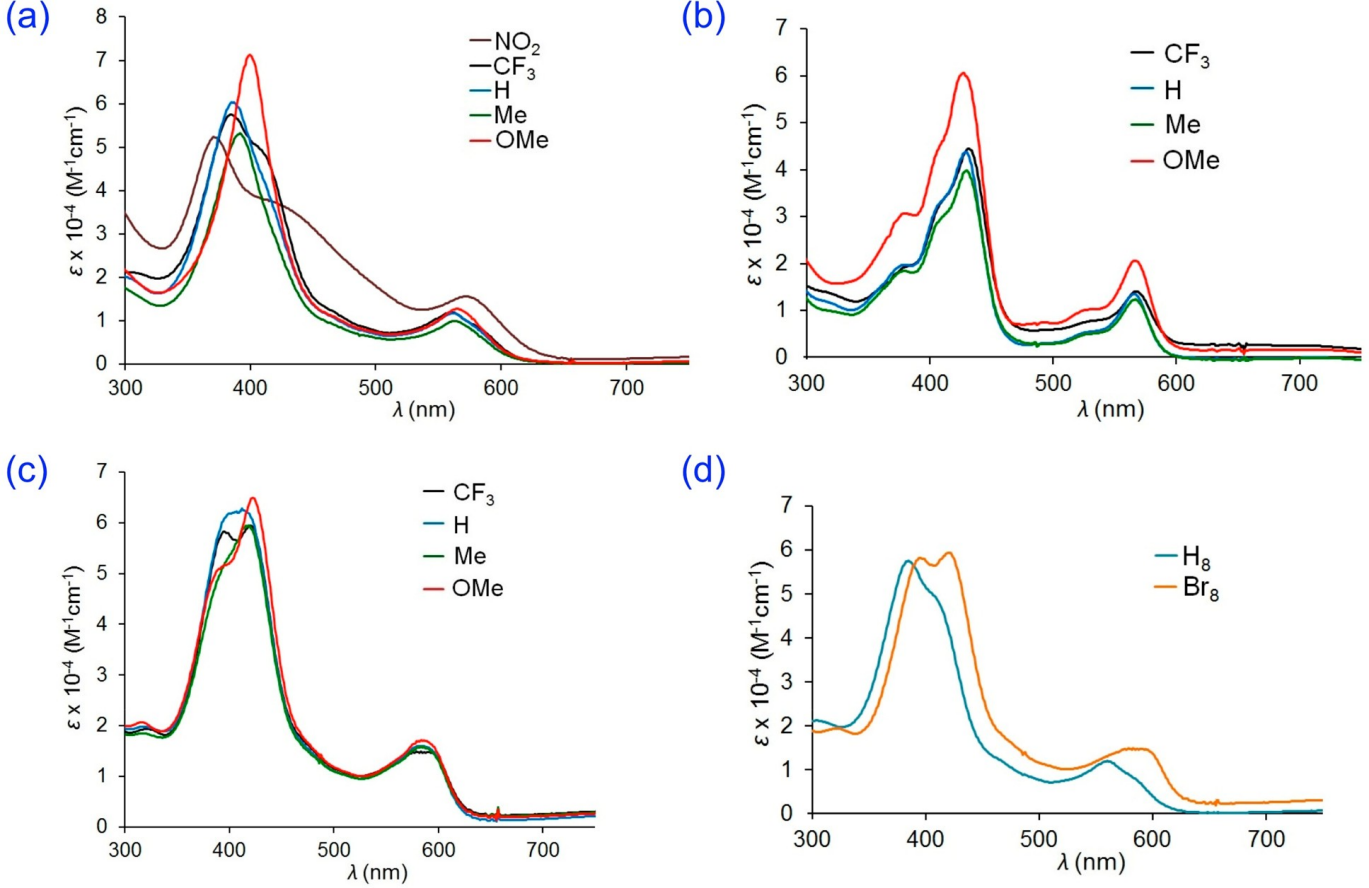
UV–vis spectra in dichloromethane for (a) Co[T*p*XPC](PPh_3_), (b) Rh[T*p*XPC](PPh_3_), (c) Co[Br_8_T*p*XPC](PPh_3_), and (d) Co[T*p*CF_3_PC](PPh_3_) and Co[Br_8_T*p*CF_3_PC](PPh_3_). Reproduced from ref (67). Copyright 2017 American Chemical Society.

**Figure 19:**
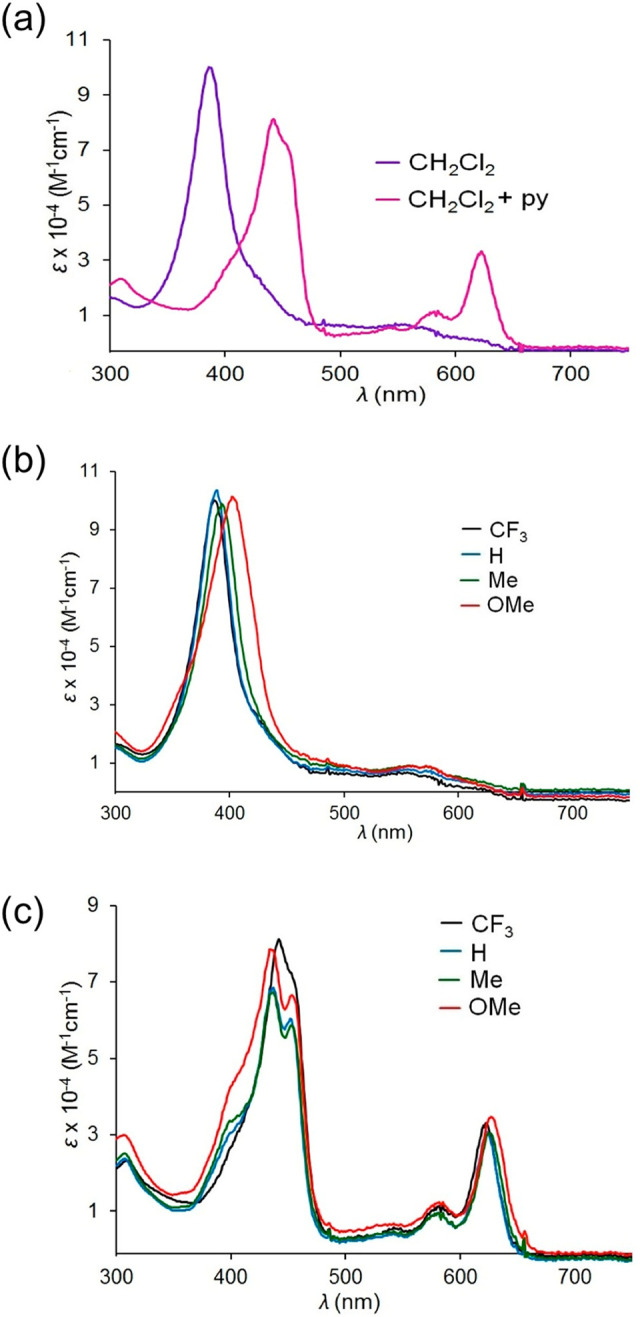
UV–vis spectra of (a) Co[T*p*CF_3_PC](py)_2_, (b) the Co[T*p*XPC](py)_2_ series in CH_2_Cl_2_, and (c) the Co[T*p*XPC](py)_2_ series in CH_2_Cl_2_ with 0.5% pyridine. Reproduced from ref (67). Copyright 2017 American Chemical Society.

One of our more surprising findings in recent years is that the five-coordinate Co[T*p*XPC](PPh_3_)^80^ (Figure 18) and Co[T*p*XPC](py)^81^ (Figure 19) series do not involve classic low-spin Co(III) centers but are best described as Co^II^-corrole^•2–^. Again, hypercorrole spectra with substituent-sensitive Soret maxima provided the first clue, which was subsequently augmented with several other lines of evidence. In contrast, the six-coordinate Co[T*p*XPC](py)_2_ series gives substituent-insensitive Soret maxima and are best thought of as genuine low-spin Co(III) complexes.^81^ In nonpolar solvents such as dichloromethane, however, one of the pyridine ligands falls off and the resulting solutions, in which the main species is Co[T*p*XPC](py), exhibit substituent-sensitive Soret maxima (Figure 19). Worth noting in this connection is that the Rh[T*p*XPC](PPh_3_)^81^ series involves innocent corrole macrocycles.

### 4.4 The Coinage Metals

**Figure 20:**
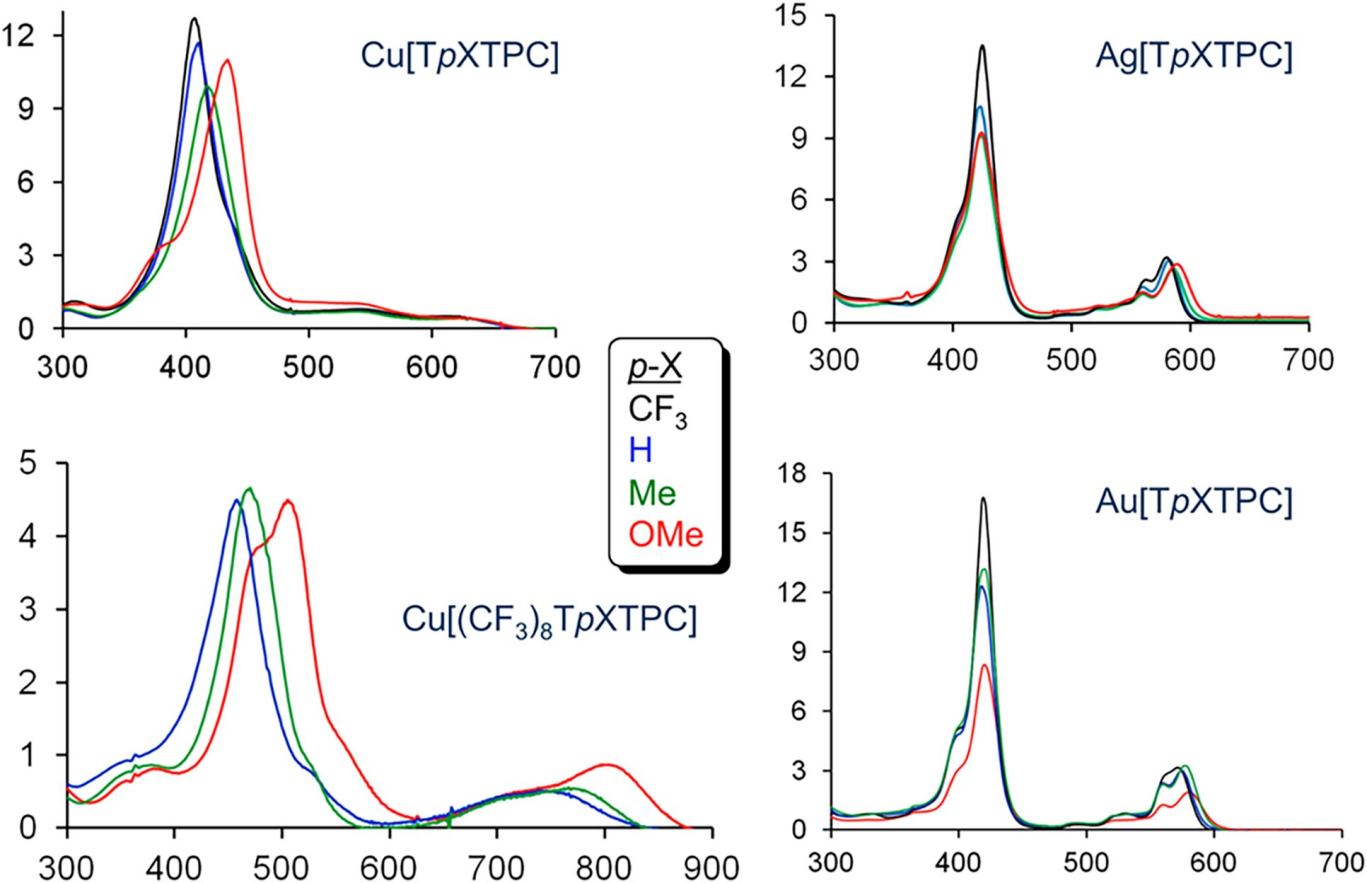
Electronic absorption spectra for M[T*p*XPC] derivatives, where M = Cu, Ag, and Au and X = CF_3_, H, Me, and OMe (color-coded as shown in the inset), and for Cu[(CF_3_)_8_T*p*XPC]. Reproduced from ref (67). Copyright 2017 American Chemical Society.

Among copper corroles,^82−84^ both the simple triarylcorrole series Cu[T*p*XPC] and the β-substituted series Cu[Br_8_T*p*XPC] and Cu[(CF_3_)_8_T*p*XPC] exhibit substituent sensitive Soret maxima (Figure 20), indicative of a Cu^II^-corrole^•2–^ description. Gold triarylcorroles, in sharp contrast, do not exhibit such substituent sensitivity, which indicates (on the basis of other additional lines of evidence) an innocent Au^III^-corrole^3–^ electronic description.^85−87^ This difference manifests itself most dramatically in the structures of isoelectronic coinage metal corroles: while Au corroles are planar, Cu corroles, uniquely among metallocorroles, are inherently saddled. The saddled conformation is associated with a Cu(d_*x*^2^–*y*^2^_)-corrole(π) orbital interaction (depicted in Figure 15), which allows part of the corrole(π) electron density to flow into the formally empty Cu(d_*x*^2^–*y*^2^_) orbital, resulting in an overall Cu^II^-corrole^•2–^ description. In the Au case, the relativistically destabilized 5d_*x*^2^–*y*^2^_ orbital is too high in energy to engage in a similar interaction, explaining both the substituent-insensitive Soret bands in the Au[T*p*XPC] series and the planar macrocycle geometries.^86,87^

Silver corroles are special in this regard. While the Ag[T*p*XPC] series exhibits essentially planar macrocycles and substituent-insensitive Soret maxima (like Au[T*p*XPC], see Figure 20), the more sterically hindered Ag[Br_8_T*p*XPC] series exhibits strongly saddled macrocycles and substituent-sensitive Soret maxima (like their Cu counterparts). The observations suggest that while the Ag[T*p*XPC] series is essentially innocent, the Ag[Br_8_T*p*XPC] series is noninnocent.^87^

### 4.5 Platinum Corroles

**Figure 21:**
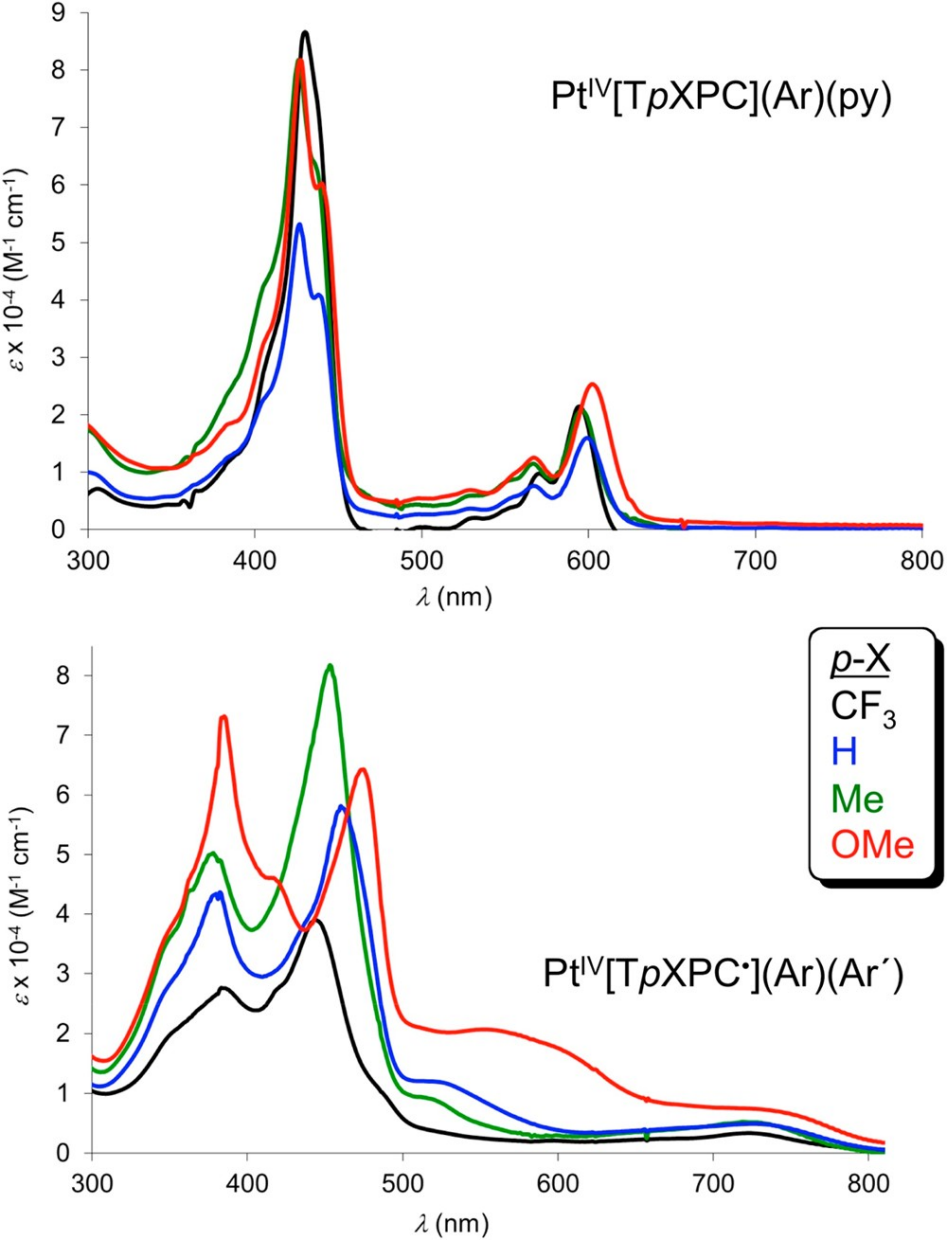
Electronic absorption spectra of the Pt(IV) (top) and oxidized Pt(IV) (bottom) series. Adapted from ref (89). Copyright 2018 American Chemical Society.

A series of neutral, paramagnetic complexes Pt[T*p*XPC](Ar)(Ar′) exemplify some of the best examples of uncoupled corrole radicals.^88^ The complexes, which may be described as Pt^IV^[T*p*XPC^•^](Ar)(Ar′), exhibit hypercorrole spectra with prominent split Soret bands in which the main visible peak is strongly substituent-dependent (Figure 21). In contrast, the true Pt(IV) series Pt[T*p*XPC](Ar)(py) exhibits much sharper, slightly split Soret bands, whose positions are essentially substituent-independent.^89^ These probably also have some hyper character, but theoretical assignments are still lacking.

## 5 Conclusions and Prospects

We have presented a contemporary and somewhat personal perspective of hyperporphyrin spectra by focusing on two major classes of compounds: centrally protonated *meso*-tetraarylporphyrins and noninnocent metallotriarylcorroles. Classic p-type and d-type hyperporphyrins have also been briefly covered, while a discussion of spectral redshifts due to conjugating β-substituents (such as halogen, alkoxy, alkylthio, amino, etc.) has been deferred for a different occasion.

Hyperporphyrin spectra can reflect various types of charge transfer transitions—MLCT, LMCT, LLCT, and combinations thereof. The term thus appears to unite a class of related electronic phenomena and a rather diverse menagerie of molecules that exhibit them. Because of the importance of porphyrins as optoelectronic materials, the ability to create hyperporphyrin effects by simple structural perturbations such as the complexation of certain elements, certain types of peripheral substituents, and acid–base reactions may be a particularly useful paradigm. Compared with Gouterman’s days, there are many new approaches to the design of NIR-absorbing and emitting dyes, such as porphyrin ring reduction,^90,91^ loss of aromaticity,^92,93^ and especially porphyrinoids with extended conjugations (e.g., nanographenes).^94^ In spite of the plethora of options, the hyperporphyrin paradigm remains attractive on account of its practical simplicity. Below are some musings on how the concept can serve as a creative force in both fundamental and application-oriented areas.

Let us consider hemes and their model compounds, including metallocorroles, that exhibit hyperporphyrin spectra. A deeper understanding of the spectra may improve our understanding of their reactivity and potentially facilitate their deployment as catalysts and therapeutics (e.g., as antioxidants for disorders mediated by oxidative stress). Such an understanding may be further enhanced by MCD^95^ and XAS spectroscopy.^76^ Many such studies of “electronic structure contributions to reactivity” have been reported by Solomon et al., but only rarely for heme and porphyrin-type systems.^96,97^ Such studies would be a welcome addition to the heme and metalloporphyrin literature.

**Figure 22:**
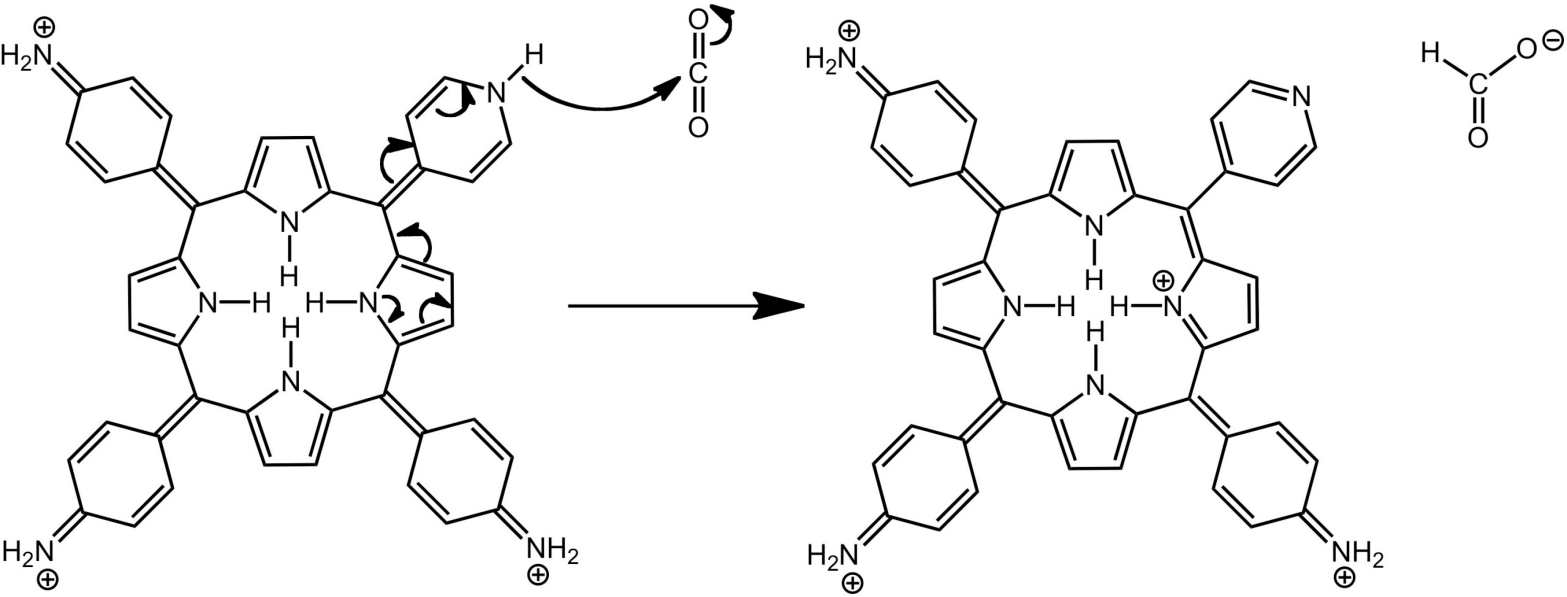
Speculative use of a Type D hyperporphyrin as a hydride donor. Reproduced from ref (35). Copyright 2014 American Chemical Society.

A significant amount of interest in porphyrins centers around their potential application to solar energy conversion, analogous to the role of chlorophyll in photosynthesis. A push–pull charge transfer motif has been at the heart of many synthetic porphyrins designed for this purpose.^98−100^ Thus, some of the most efficient photosensitizers employed in dye-sensitized solar cells are porphyrins with an *N*,*N*-diarylamino *meso* substituent and a carboxyphenyl anchoring group.^101,102^ The unique Type D resonance form illustrated earlier has been suggested as a potential two-electron hydride donor by analogy to the NADH/NAD^+^ couple (Figure 22).^35^ In this case, the N–H bond of a protonated pyridine is suggested as a hydride donor, leaving behind an oxidized porphyrin. Such a process might be photochemically induced.

In spite of their attractive spectra for solar absorption, typically extending into the far red, hyperporphyrin systems based on protonated tetraphenylporphyrins have not been directly examined as photosensitizers. Presumably, because it is necessary to generate such systems under strongly acidic conditions, they have been little used for solar sensitization, as well as biomedical applications such as in photodynamic therapy. That said, protonated tetraphenylporphyrins *have* been used as sensors for gases such as ammonia, hydrogen sulfide, and sulfur dioxide.^103,104^

As a final example, 5d metalloporphyrins and metallocorroles, which are of great interest as triplet photosensitizers, might be considered.^24,105−107^ Gouterman himself devoted the latter part of his career to developing phosphorescent Pt porphyrins as pressure-sensitive paints for airplane wings.^108,109^ In one of our own laboratories, we are developing 5d metallocorroles as dyes for oxygen sensing, photodynamic therapy, and triplet–triplet annihilation upconversion.^24^ Although several of these complexes are actually of the hypso type, the use of suitable substituents might significantly redshift key absorption and emission features.^10^ In such a case, the hypso/hyper distinction might become moot. We hope to illustrate such applications of the hyperporphyrin concept by synthesizing new classes of 5d metallocorrole-based photosensitizers.

## References

[ref1] Handbook of Porphyrin Science, Vol. 1–45; Kadish K. M., Smith K. M., Guilard R., Eds.; World Scientific, 2010.

[ref200] Sessler J. L.; Gross Z.; Furuta H. Introduction: Expanded, Contracted, and Isomeric Porphyrins. Chem. Rev. 2017, 117, 2201–2202. 10.1021/acs.chemrev.7b00036. 28222607

[ref2] Ghosh A. An Exemplary Gay Scientist and Mentor: Martin Gouterman (1931–2020). Angew. Chem. Int. Ed. 2021, 60, 9760–9770. 10.1002/anie.202012840. 33689208

[ref3] Gouterman M. Spectra of porphyrins. J. Mol. Spectrosc. 1961, 6, 138–163. 10.1016/0022-2852(61)90236-3.

[ref4] Gouterman M.; Wagniére G. H.; Snyder L. C. Spectra of Porphyrins. Part II. Four-Orbital Model. J. Mol. Spectrosc. 1963, 11, 108–115. 10.1016/0022-2852(63)90011-0.

[ref5] Gouterman M. Optical Spectra and Electronic Structure of Porphyrins and Related Rings. In The Porphyrins, Vol. III, (Part A), ; Dolphin D., Ed.; Academic Press: New York, 1978; pp 1–165.

[ref6] The calculated data on Al^III^[TPC](NH_3_)_2_ have been compared to experimental data on Al^III^[TPFPC](py)_2_: Mahammed A.; Gross Z. Aluminum Corrolin, A Novel Chlorophyll Analogue. J. Inorg. Biochem 2002, 88, 305–309. 10.1016/S0162-0134(01)00373-7. 11897344

[ref7] Bendix J.; Dmochowski I. J.; Gray H. B.; Mahammed A.; Simkhovich L.; Gross Z. Structural, Electrochemical, and Photophysical Properties of Gallium(III) 5,10,15-Tris(pentafluorophenyl)corrole. Angew. Chem. Int. Ed 2000, 39, 4048–4051. 10.1002/1521-3773(20001117)39:22<4048::AID-ANIE4048>3.0.CO;2-7. 11093200

[ref8] Kowalska D.; Liu X.; Tripathy U.; Mahammed A.; Gross Z.; Hirayama S.; Steer R. P. Ground- and Excited-State Dynamics of Aluminum and Gallium Corroles. Inorg. Chem. 2009, 48, 2670–2676. 10.1021/ic900056n. 19267510

[ref9] Ghosh A.; Wondimagegn T.; Parusel A. B. J. Electronic Structure of Gallium, Copper, and Nickel Complexes of Corrole. High-Valent Transition Metal Centers Versus Noninnocent Ligands. J. Am. Chem. Soc. 2000, 122, 5100–5104. 10.1021/ja9943243.

[ref10] Ghosh A.; Conradie J. The Dog That Didn’t Bark: A New Interpretation of Hypsoporphyrin Spectra and the Question of Hypsocorroles. J. Phys. Chem. A 2021, 125, 9962–9968. 10.1021/acs.jpca.1c08425. 34762440 PMC8630793

[ref11] Hanson L. K.; Eaton W. A.; Sligar S. G.; Gunsalus I. C.; Gouterman M.; Connell C. R. Origin of the anomalous Soret spectra of carboxycytochrome P-450. J. Am. Chem. Soc. 1976, 98, 2672–2674. 10.1021/ja00425a050. 1262660

[ref12] Sayer P.; Gouterman M.; Connell C. R. Metalloid Porphyrins and Phthalocyanines. Acc. Chem. Res. 1982, 15, 73–79. 10.1021/ar00075a002.

[ref13] Buchler J. W. Synthesis and Properties of Metalloporphyrins. In The Porphyrins, Vol. I, (Part A), ; Dolphin D., Ed.; Academic Press: New York, 1978; pp 389–483.

[ref14] Cissell J. A.; Vaid T. P.; Yap G. P. A. Reversible Oxidation State Change in Germanium(tetraphenylporphyrin) Induced by a Dative Ligand: Aromatic Ge^II^(TPP) and Antiaromatic Ge^IV^(TPP)(pyridine)_2_. J. Am. Chem. Soc. 2007, 129, 7841–7847. 10.1021/ja070794i. 17550248

[ref15] Barbe J. M.; Ratti C.; Richard P.; Lecomte C.; Gerardin R.; Guilard R. Tin(II) porphyrins: synthesis and spectroscopic properties of a series of divalent tin porphyrins. X-ray crystal structure of (2,3,7,8,12,13,17,18-octaethylporphinato)tin(II). Inorg. Chem. 1990, 29, 4126–4130. 10.1021/ic00345a043.

[ref16] Arnold D. P.; Blok J. The coordination chemistry of tin porphyrin complexes. Coord. Chem. Rev. 2004, 248, 299–319. 10.1016/j.ccr.2004.01.004.

[ref17] Luobeznova I.; Raizman M.; Goldberg I.; Gross Z. Synthesis and Full Characterization of Molybdenum and Antimony Corroles and Utilization of the Latter Complexes as Very Efficient Catalysts for Highly Selective Aerobic Oxygenation Reactions. Inorg. Chem. 2006, 45, 386–394. 10.1021/ic051483g. 16390079

[ref18] Lemon C. M.; Maher A. G.; Mazzotti A. R.; Powers D. C.; Gonzalez M. I.; Nocera D. G. Multielectron C–H photoactivation with an Sb(V) oxo corrole. Chem. Commun. 2020, 56, 5247–5250. 10.1039/C9CC09892E. 32270146

[ref19] Mondal S.; Garai A.; Naik P. K.; Adha J. K.; Kar S. Synthesis and characterization of antimony(V)-oxo corrole complexes. Inorg. Chim. Acta 2020, 501, 119300 10.1016/j.ica.2019.119300.

[ref20] Cissell J. A.; Vaid T. P.; Rheingold A. L. An Antiaromatic Porphyrin Complex: Tetraphenylporphyrinato(silicon)(L)_2_ (L = THF or Pyridine). J. Am. Chem. Soc. 2005, 127, 12212–12213. 10.1021/ja0544713. 16131185

[ref21] Weiss A.; Hodgson M. C.; Boyd P. D. W.; Siebert W.; Brothers P. J. Diboryl and Diboranyl Porphyrin Complexes: Synthesis, Structural Motifs, and Redox Chemistry: Diborenyl Porphyrin or Diboranyl Isophlorin?. Chem. Eur. J. 2007, 13, 5982–5993. 10.1002/chem.200700046. 17570718

[ref22] Conradie J.; Brothers P. J.; Ghosh A. Main-Group-Element Isophlorin Complexes Revisited: The Question of a Subvalent Central Atom. Inorg. Chem. 2019, 58, 4634–4640. 10.1021/acs.inorgchem.9b00201. 30874434

[ref23] Parkin G. Valence, Oxidation Number, and Formal Charge: Three Related but Fundamentally Different Concepts. J. Chem. Educ. 2006, 83, 791–799. 10.1021/ed083p791.

[ref24] Alemayehu A. B.; Thomas K. E.; Einrem R. F.; Ghosh A. The Story of 5d Metallocorroles: From Metal–Ligand Misfits to New Building Blocks for Cancer Phototherapeutics. Acc. Chem. Res. 2021, 54, 3095–3107. 10.1021/acs.accounts.1c00290. 34297542 PMC8382219

[ref25] Hanson L. K.; Eaton W. A.; Sligar S. G.; Gunsalus I. C.; Gouterman M.; Connell C. R. Origin of the anomalous Soret spectra of carboxycytochrome P-450. J. Am. Chem. Soc. 1976, 98, 2672–2674. 10.1021/ja00425a050. 1262660

[ref26] Hunt A. P.; Lehnert N. The Thiolate Trans Effect in Heme {FeNO}^6^ Complexes and Beyond: Insight into the Nature of the Push Effect. Inorg. Chem. 2019, 58, 11317–11332. 10.1021/acs.inorgchem.9b00091. 30912445

[ref27] Kühnel K.; Derat E.; Terner J.; Shaik S.; Schlichting I. Structure and quantum chemical characterization of chloroperoxidase compound 0, a common reaction intermediate of diverse heme enzymes. Proc. Nat. Acad. Soc. USA 2007, 104, 99–104. 10.1073/pnas.0606285103. PMC1765485 17190816

[ref28] Green M. T.; Dawson J. H.; Gray H. B. Oxoiron(IV) in Chloroperoxidase Compound II Is Basic: Implications for P450 Chemistry. Science 2004, 304, 1653–1656. 10.1126/science.1096897. 15192224

[ref29] Rittle J.; Green M. T. Cytochrome P450 Compound I: Capture, Characterization, and C-H Bond Activation Kinetics. Science 2010, 330, 933–937. 10.1126/science.1193478. 21071661

[ref30] Yosca T. H.; Rittle J.; Krest C. M.; Onderko E. L.; Silakov A.; Calixto J. C.; Behan R. K.; Green M. T. Iron(IV)hydroxide pKa and the Role of Thiolate Ligation in C–H Bond Activation by Cytochrome P450. Science 2013, 342, 825–829. 10.1126/science.1244373. 24233717 PMC4299822

[ref31] Yosca T. H.; Ledray A. P.; Ngo J.; Green M. T. A new look at the role of thiolate ligation in cytochrome P450. J. Biol. Inorg. Chem. 2017, 22, 209–220. 10.1007/s00775-016-1430-3. 28091754 PMC5640440

[ref32] Yosca T. H.; Langston M. C.; Krest C. M.; Onderko E. L.; Grove T. L.; Livada J.; Green M. T. Spectroscopic Investigations of Catalase Compound II: Characterization of an Iron(IV) Hydroxide Intermediate in a Non-thiolate-Ligated Heme Enzyme. J. Am. Chem. Soc. 2016, 138, 16016–16023. 10.1021/jacs.6b09693. 27960340 PMC5987761

[ref33] Ojadi E. C. A.; Linschitz H.; Gouterman M.; Walter R. I.; Lindsey J. S.; Wagner R. W.; Droupadi P. R.; Wang W. Sequential Protonation of *meso*-(*p*-(Dimethylamino)phenyl)porphyrins: Charge-Transfer Excited States Producing Hyperporphyrins. J. Phys. Chem. 1993, 97, 13192–13197. 10.1021/j100152a025.

[ref34] Rudine A. B.; DelFatti B. D.; Wamser C. C. Spectroscopy of Protonated Tetraphenylporphyrins with Amino/Carbomethoxy Substituents: Hyperporphyrin Effects and Evidence for a Monoprotonated Porphyrin. J. Org. Chem. 2013, 78, 6040–6049. 10.1021/jo400742f. 23663204

[ref35] Wang C.; Wamser C. C. Hyperporphyrin Effects in the Spectroscopy of Protonated Porphyrins with 4-Aminophenyl and 4-Pyridyl *Meso* Substituents. J. Phys. Chem. A 2014, 118, 3605–3615. 10.1021/jp501398g. 24754721

[ref36] Fleischer E. Structure of porphyrins and metalloporphyrins. Acc. Chem. Res. 1970, 3, 105–112. 10.1021/ar50027a004.

[ref37] Kadish K. M.; Morrison M. M.; Constant L. A.; Dickens L.; Davis D. G. A study of solvent and substituent effects on the redox potentials and electron-transfer rate constants of substituted iron *meso*-tetraphenylporphyrins. J. Am. Chem. Soc. 1976, 98, 8387–8390. 10.1021/ja00442a013. 993529

[ref38] Kadish K. M.; Morrison M. M. Substituent Effects on the Oxidation-Reduction Reactions of Nickel Para-Substituted Tetraphenylporphyrin in Nonaqueous Media. Inorg. Chem. 1976, 15, 980–982. 10.1021/ic50158a055. 1262646

[ref39] Ransdell R. A.; Wamser C. C. Solvent and substituent effects on the redox properties of free-base tetraphenylporphyrins in DMSO and aqueous DMSO. J. Phys. Chem. 1992, 96, 10572–10575. 10.1021/j100204a081.

[ref40] Weinkauf J. R.; Cooper S. W.; Schweiger A.; Wamser C. C. Substituent and Solvent Effects on the Hyperporphyrin Spectra of Diprotonated Tetraphenylporphyrins. J. Phys. Chem. A 2003, 107, 3486–3496. 10.1021/jp022046f.

[ref41] Meot-Ner M.; Adler A. D. Substituent effects in noncoplanar π systems. ms-Porphins. J. Am. Chem. Soc. 1975, 97, 5107–5111. 10.1021/ja00851a014.

[ref42] Stone A.; Fleischer E. B. The Molecular and Crystal Structure of Porphyrin Diacids. J. Am. Chem. Soc. 1968, 90, 2735–2748. 10.1021/ja01013a001.

[ref43] Cheng B.; Munro O. Q.; Marques H. M.; Scheidt W. R. An Analysis of Porphyrin Molecular Flexibility: Use of Porphyrin Diacids. J. Am. Chem. Soc. 1997, 119, 10732–10742. 10.1021/ja9716214.

[ref44] Wasbotten I. H.; Conradie J.; Ghosh A. Electronic Absorption and Resonance Raman Signatures of Hyperporphyrins and Nonplanar Porphyrins. J. Phys. Chem. B 2003, 107, 3613–3623. 10.1021/jp014417i.

[ref45] Hibbert F.; Hunte K. P. P. Kinetic and equilibrium studies of the protonation of meso-tetraphenylporphyrin in dimethyl sulfoxide–water. J. Chem. Soc., Perkin Trans. 1977, 2, 1624–1628. 10.1039/P29770001624.

[ref46] Zurita A.; Duran A.; Ribó J. M.; El-Hachemi Z.; Crusats J. Hyperporphyrin effects extended into a J-aggregate supramolecular structure in water. RSC Adv. 2017, 7, 3353–3357. 10.1039/C6RA27441B.

[ref47] Vitasovic M.; Gouterman M.; Linschitz H. Calculations on the origin of hyperporphyrin spectra in *meso*-(dimethylaminophenyl) porphyrins. J. Porphyrins Pthalocyanines 2001, 5, 191–197. 10.1002/jpp.309.

[ref48] Walter R. I.; Ojadi E. C. A.; Linschitz H. A Proton NMR Study of the Reactions with Acid of meso-Tetraphenylporphyrins with Various Numbers of 4-Dimethylamino Groups. J. Phys. Chem. 1993, 97, 13308–13312. 10.1021/j100152a039.

[ref49] Wang C.; Wamser C. C. NMR Study of Hyperporphyrin Effects in the Protonations of Porphyrins with 4-Aminophenyl and 4-Pyridyl Meso Substituents. J. Org. Chem. 2015, 80 (15), 7351–7359. 10.1021/acs.joc.5b00690. 26168319

[ref50] Guo H.; Jiang J.; Shi Y.; Wang Y.; Dong S. Solvent effects on spectrophotometric titrations and vibrational spectroscopy of 5,10,15-triphenyl-20-(4-hydroxyphenyl)porphyrin in aqueous DMF. Spectrochim. Acta, Part A 2007, 67, 166–171. 10.1016/j.saa.2006.06.040. 17267277

[ref51] Conradie J.; Wamser C. C.; Ghosh A. Understanding Hyperporphyrin Spectra: TDDFT Calculations on Diprotonated Tetrakis(*p*-aminophenyl)porphyrin. J. Phys. Chem. A 2021, 125, 9953–9961. 10.1021/acs.jpca.1c06621. 34714662 PMC8630795

[ref52] Milgrom L. R.; Hill J. P.; Yahioglu G. Facile aerial oxidation of a porphyrin. Part 18. N-alkylation of the oxidised product derived from *Meso*-tetrakis(3,5-di-t-butyl-4-hydroxyphenyl)porphyrin. J. Heterocycl. Chem. 1995, 32, 97–101. 10.1002/jhet.5570320117.

[ref53] Guo H.; Jiang J.; Shi Y.; Wang Y.; Liu J.; Dong S. UV-Vis Spectrophotometric Titrations and Vibrational Spectroscopic Characterization of meso-(*p*-Hydroxyphenyl)porphyrins. J. Phys. Chem. B 2004, 108, 10185–10191. 10.1021/jp048981f.

[ref54] Guo H.; Jiang J.; Shi Y.; Wang Y.; Wang Y.; Dong S. Sequential Deprotonation of meso-(*p*-Hydroxyphenyl)porphyrins in DMF: From Hyperporphyrins to Sodium Porphyrin Complexes. J. Phys. Chem. B 2006, 110, 587–594. 10.1021/jp0523827. 16471571

[ref55] Hill J. P.; Hewitt I. J.; Anson C. E.; Powell A. K.; McCarty A. L.; Karr P. A.; Zandler M. E.; D’Souza F. Highly Nonplanar, Electron Deficient, N-Substituted tetra-Oxocyclohexadienylidene Porphyrinogens: Structural, Computational, and Electrochemical Investigations. J. Org. Chem. 2004, 69, 5861–5869. 10.1021/jo049401d. 15373471

[ref56] Chahal M. K.; Payne D. T.; Matsushita Y.; Labuta J.; Ariga K.; Hill J. P. Molecular Engineering of β-Substituted Oxoporphyrinogens for Hydrogen-Bond Donor Catalysis. Eur. J. Org. Chem. 2020, 2020, 82–90. 10.1002/ejoc.201901706.

[ref57] Goldberg P. K.; Pundsack T. J.; Splan K. E. Photophysical Investigation of Neutral and Diprotonated Free-Base Bis(Arylethynyl)porphyrins. J. Phys. Chem. A 2011, 115, 10452–10460. 10.1021/jp205309f. 21793565

[ref58] Evens K. K.; Kaplan K. E. Spectroscopic characterization of free-base hydroxy(arylethynyl)porphyrins in acidic and basic media. J. Porphyrins Phthalocyanines 2017, 21, 680–691. 10.1142/S1088424617500675.

[ref59] Gündüz N.; Gündüz T.; Hayvalı M. Titrations in non-aqueous media: potentiometric investigation of symmetrical and unsymmetrical tetra-aryl porphyrins with 4-nitrophenyl and 4-aminophenyl substituents in nitrobenzene sovent. Talanta 1999, 48, 71–79. 10.1016/S0039-9140(98)00222-7. 18967445

[ref60] Hambright P.; Fleischer E. B. The Acid-Base Equilibria, Kinetics of Copper Ion Incorporation, and Acid-Catalyzed Zinc Ion Displacement from the Water-Soluble Porphyrin *α,β,γ,δ*-Tetra(4-N-methylpyridyl)porphine. Inorg. Chem. 1970, 9, 1757–1761. 10.1021/ic50089a030.

[ref61] Pasternack R. F.; Huber P. R.; Boyd P.; Engasser G.; Francesconi L.; Gibbs E.; Fasella P.; Venturo G. C.; Hinds L. deC. On the Aggregation of Meso-Substituted Water-Soluble Porphyrins. J. Am. Chem. Soc. 1972, 94, 4511–4517. 10.1021/ja00768a016. 5036163

[ref62] Santiago P. S.; Gandini S. C. M.; Moreira L. M.; Tabak M. Interaction of cationic water-soluble *meso*-tetrakis(4-N-methylpyridiniumyl)porphyrin (TMPyP) with ionic and nonionic micelles: aggregation and binding. J. Porphyrins Phthalocyanines 2008, 12, 942–952. 10.1142/S1088424608000327.

[ref63] Meng G. G.; James B. R.; Skov K. A.; Korbelik M. Porphyrin chemistry pertaining to the design of anti-cancer drugs; part 2, the synthesis and in vitro tests of water-soluble porphyrins containing, in the meso positions, the functional groups: 4-methylpyridinium, or 4-sulfonatophenyl, in combination with phenyl, 4-pyridyl, 4-nitrophenyl, or 4-aminophenyl. Can. J. Chem. 1994, 72, 2447–2457. 10.1139/v94-311.

[ref64] LeCours S. M.; Philips C. M.; De Paula J. C.; Therien M. J. Synthesis, Transient Absorption, and Transient Resonance Raman Spectroscopy of Novel Electron Donor-Acceptor Complexes: [5,15-Bis[(4′-nitrophenyl)ethynyl]-10,20- diphenylporphinato]copper(II) and [5-[[4′-(Dimethylamino)phenyl]ethynyl]-15-[(4″-nitrophenyl)ethynyl]-10,20-diphenylporphinato]copper(II). J. Am. Chem. Soc. 1997, 119, 12578–12589. 10.1021/ja964436j. 28590731

[ref65] Walter M. G.; Wamser C. C. Synthesis and Characterization of Electropolymerized Nanostructured Aminophenylporphyrin Films. J. Phys. Chem. C 2010, 114, 7563–7574. 10.1021/jp910016h.

[ref66] Day N. U.; Walter M. G.; Wamser C. C. Preparations and Electrochemical Characterizations of Conductive Porphyrin Polymers. J. Phys. Chem. C 2015, 119, 17378–17388. 10.1021/acs.jpcc.5b02628.

[ref67] Ghosh A. Electronic Structure of Corrole Derivatives: Insights from Molecular Structures, Spectroscopy, Electrochemistry, and Quantum Chemical Calculations. Chem. Rev. 2017, 117, 3798–3881. 10.1021/acs.chemrev.6b00590. 28191934

[ref68] Mahammed A.; Weaver J. J.; Gray H. B.; Abdelas M.; Gross Z. How Acidic Are Corroles and Why?. Tetrahedron Lett. 2003, 44, 2077–2079. 10.1016/S0040-4039(03)00174-6.

[ref201] Jimenez H. R.; Julve M.; Faus J. A Solution Study of the Protonation and Deprotonation Equilibria of 5,10,15,20-Tetra(*p*-sulphonatophenyl)porphyrin. Stability Constants of its Magnesium(II), Copper(II) and Zinc(II) Complexes. J. Chem. Soc., Dalton Trans. 1991, 1945–1949. 10.1039/DT9910001945.

[ref69] Song Y.; Fang Y.; Ou Z.; Capar J.; Wang C.; Conradie J.; Thomas K. E.; Wamser C. C.; Ghosh A.; Kadish K. M. Influence of β-octabromination on free-base triarylcorroles: Electrochemistry and protonation-deprotonation reactions in nonaqueous media. J. Porphyrins Phthalocyanines 2017, 21, 633–645. 10.1142/S1088424617500602.

[ref70] Shen J.; Ou Z.; Shao J.; Galezowski M.; Gryko D. T.; Kadish K. M. Free-Base Corroles: Determination of Deprotonation Constants in Non-Aqueous Media. J. Porphyrins Phthalocyanines 2007, 11, 269–276. 10.1142/S1088424607000321.

[ref71] Thomassen I. K.; Ghosh A. Protonation-Induced Hyperporphyrin Spectra of *meso*-Aminophenylcorroles. ACS Omega 2020, 5, 9023–9030. 10.1021/acsomega.0c01068. 32337467 PMC7178790

[ref72] Ganguly S.; Ghosh A. Seven Clues to Ligand Noninnocence: The Metallocorrole Paradigm. Acc. Chem. Res. 2019, 52, 2003–2014. 10.1021/acs.accounts.9b00115. 31243969

[ref73] Alemayehu A. B.; Conradie J.; Ghosh A. A First TDDFT Study of Metallocorrole Electronic Spectra: Copper *meso*-Triarylcorroles Exhibit *Hyper* Spectra. Eur. J. Inorg. Chem. 2011, 12, 1857–1864. 10.1002/ejic.201001026.

[ref74] Ganguly S.; McCormick L. J.; Conradie J.; Gagnon K. J.; Sarangi R.; Ghosh A. Electronic Structure of Manganese Corroles Revisited: X-ray Structures, Optical and X-ray Absorption Spectroscopies, and Electrochemistry as Probes of Ligand Noninnocence. Inorg. Chem. 2018, 57, 9656–9669. 10.1021/acs.inorgchem.8b00537. 29873229

[ref75] Steene E.; Wondimagegn T.; Ghosh A. Electrochemical and Electronic Absorption Spectroscopic Studies of Substituent Effects in Iron(IV) and Manganese(IV) Corroles. Do the Compounds Feature High-Valent Metal Centers or Noninnocent Corrole Ligands? Implications for Peroxidase Compound I and II Intermediates. J. Phys. Chem. B 2001, 105, 11406–11413. 10.1021/jp012037r.

[ref76] Ganguly S.; Giles L. J.; Thomas K. E.; Sarangi R.; Ghosh A. Ligand Noninnocence in Iron Corroles: Insights from Optical and X-ray Absorption Spectroscopies and Electrochemical Redox Potentials. Chem. Eur. J. 2017, 23, 15098–15106. 10.1002/chem.201702621. 28845891 PMC5710759

[ref77] Simkhovich L.; Mahammed A.; Goldberg I.; Gross Z. Synthesis and Characterization of Germanium, Tin, Phosphorus, Iron, and Rhodium Complexes of Tris(pentafluorophenyl)corrole, and the Utilization of the Iron and Rhodium Corroles as Cyclopropanation Catalysts. Chem. Eur. J. 2001, 7, 1041–1055. 10.1002/1521-3765(20010302)7:5<1041::AID-CHEM1041>3.0.CO;2-8. 11303864

[ref78] Vazquez-Lima H.; Norheim H. K.; Einrem R. F.; Ghosh A. Cryptic Noninnocence: FeNO Corroles in a New Light. Dalton Trans 2015, 44, 10146–10151. 10.1039/C5DT01495F. 25978788

[ref79] Ganguly S.; Vazquez-Lima H.; Ghosh A. Wolves in Sheep’s Clothing: μ-Oxo-Diiron Corroles Revisited. Chem. Eur. J. 2016, 22, 10336–10340. 10.1002/chem.201601062. 27333259

[ref80] Ganguly S.; Renz D.; Giles L. J.; Gagnon K. J.; McCormick L. J.; Conradie J.; Sarangi R.; Ghosh A. Cobalt- and Rhodium-Corrole-Triphenylphosphine Complexes Revisited: the Question of a Noninnocent Corrole. Inorg. Chem. 2017, 56, 14788–14800. 10.1021/acs.inorgchem.7b01828. 29210572

[ref81] Ganguly S.; Conradie J.; Bendix J.; Gagnon K. J.; McCormick L. J.; Ghosh A. Electronic Structure of Cobalt–Corrole–Pyridine Complexes: Noninnocent Five-Coordinate Co(II) Corrole–Radical States. J. Phys. Chem. A 2017, 121, 9589–9598. 10.1021/acs.jpca.7b09440. 29227102

[ref82] Wasbotten I. H.; Wondimagegn T.; Ghosh A. Electronic Absorption, Resonance Raman, and Electrochemical Studies of Planar and Saddled Copper(III) *meso*-Triarylcorroles. Highly Substituent-Sensitive Soret Bands as a Distinctive Feature of High-Valent Transition Metal Corroles. J. Am. Chem. Soc. 2002, 124, 8104–8116. 10.1021/ja0113697. 12095356

[ref83] Steene E.; Dey A.; Ghosh A. β-Octafluorocorroles. J. Am. Chem. Soc. 2003, 125, 16300–16309. 10.1021/ja021158h. 14692771

[ref84] Thomas K. E.; Wasbotten I. H.; Ghosh A. Copper β-Octakis(Trifluoromethyl)Corroles: New Paradigms for Ligand Substituent Effects in Transition Metal Complexes. Inorg. Chem. 2008, 47, 10469–10478. 10.1021/ic801101k. 18928275

[ref85] Alemayehu A. B.; Ghosh A. Gold Corroles. J. Porphyrins Phthalocyanines 2011, 15, 106–110. 10.1142/S1088424611003045.

[ref86] Thomas K. E.; Alemayehu A. B.; Conradie J.; Beavers C.; Ghosh A. Synthesis and Molecular Structure of Gold Triarylcorroles. Inorg. Chem. 2011, 50, 12844–12851. 10.1021/ic202023r. 22111600

[ref87] aThomas K. E.; Vazques-Lima H.; Fang Y.; Song Y.; Gagnon K. J.; Beavers C. M.; Kadish K. M.; Ghosh A. Ligand Noninnocence in Coinage Metal Corroles: A Silver Knife-Edge. Chem. Eur. J. 2015, 21, 16839–16847. 10.1002/chem.201502150. 26345592

[ref88] Alemayehu A. B.; Vazquez-Lima H.; Beavers C. M.; Gagnon K. J.; Bendix J.; Ghosh A. Platinum Corroles. Chem. Commun. 2014, 50, 11093–11096. 10.1039/C4CC02548B. 24911328

[ref89] Alemayehu A. B.; McCormick L. J.; Gagnon K. J.; Borisov S. M.; Ghosh A. Stable Platinum(IV) Corroles: Synthesis, Molecular Structure, and Room-Temperature Near-IR Phosphorescence. ACS Omega 2018, 3, 9360–9368. 10.1021/acsomega.8b01149. 31459069 PMC6645213

[ref90] Riahin C.; Meares A.; Esemoto N. N.; Ptaszek M.; LaScola M.; Pandala N.; Lavik E.; Yang M.; Stacey G.; Hu D.; Traeger J. C.; Orr G.; Rosenzweig Z. Hydroporphyrin-Doped Near-Infrared-Emitting Polymer Dots for Cellular Fluorescence Imaging. ACS Appl. Mater. Interfaces 2022, 14, 20790–20801. 10.1021/acsami.2c02551. 35451825 PMC9210996

[ref91] Luciano M.; Brückner C. Modifications of Porphyrins and Hydroporphyrins for Their Solubilization in Aqueous Media. Molecules 2017, 22, 980 10.3390/molecules22060980. PMC6152633 28608838

[ref92] Nardis S.; Pomarico G.; Mandoj F.; Fronczek F. R.; Smith K. M.; Paolesse R. One-pot synthesis of meso-alkyl substituted isocorroles: the reaction of a triarylcorrole with Grignard reagent. J. Porphyrins Pthalocyanines 2010, 14, 752–757. 10.1142/S1088424610002513. PMC3002075 21165169

[ref93] Foroutan-Nejad C.; Larsen S.; Conradie J.; Ghosh A. Isocorroles as Homoaromatic NIR-Absorbing Chromophores: A First Quantum Chemical Study. Sci. Rep 2018, 8, 11952 10.1038/s41598-018-29819-3. 30097587 PMC6086901

[ref94] Borissov A.; Maurya Y. K.; Moshniaha L.; Wong W.-S.; Żyła-Karwowska M.; Stępień M. Recent Advances in Heterocyclic Nanographenes and Other Polycyclic Heteroaromatic Compounds. Chem. Rev. 2022, 122, 565–788. 10.1021/acs.chemrev.1c00449. 34850633 PMC8759089

[ref95] Walker F. A. Magnetic spectroscopic (EPR, ESEEM, Mössbauer, MCD and NMR) studies of low-spin ferriheme centers and their corresponding heme proteins. Coord. Chem. Rev. 1999, 185–186, 471–534. 10.1016/S0010-8545(99)00029-6.

[ref96] Hocking R. K.; Wasinger E. C.; Yan Y.-L.; deGroot F. M. F.; Walker F. A.; Hodgson K. O.; Hedman B.; Solomon E. I. Fe L-Edge X-ray Absorption Spectroscopy of Low-Spin Heme Relative to Non-heme Fe Complexes: Delocalization of Fe d-Electrons into the Porphyrin Ligand. J. Am. Chem. Soc. 2007, 129, 113–125. 10.1021/ja065627h. 17199290 PMC2890250

[ref97] Wilson S. A.; Kroll T.; Decreau R. A.; Hocking R. K.; Lundberg M.; Hedman B.; Hodgson K. O.; Solomon E. I. Iron L-Edge X-ray Absorption Spectroscopy of Oxy-Picket Fence Porphyrin: Experimental Insight into Fe–O_2_ Bonding. J. Am. Chem. Soc. 2013, 135, 1124–1136. 10.1021/ja3103583. 23259487 PMC3614349

[ref98] Imahori H.; Kurotobi K.; Walter M. G.; Rudine A. B.; Wamser C. C. Porphyrin- and Phthalocyanine-Based Solar Cells. In Handbook of Porphyrin Science, Vol. 18; Kadish K. M., Smith K. M., Guilard R., Eds.; World Scientific: Singapore, 2012; pp 57–121.

[ref99] Walter M.; Rudine A. B.; Wamser C. C. Porphyrins and phthalocyanines in solar photovoltaic cells. J. Porphyrins Phthalocyanines 2010, 14, 759–792. 10.1142/S1088424610002689.

[ref100] Milgrom L. Synthesis of some New Tetra-arylporphyrins for Studies in Solar Energy Conversion. J. Chem. Soc. Perkin Trans. I 1983, 2535–2539. 10.1039/p19830002535.

[ref101] Bessho T.; Zakeeruddin S. M.; Yeh C.-Y.; Diau E. W.-G.; Grätzel M. Highly Efficient Mesoscopic Dye-Sensitized Solar Cells Based on Donor–Acceptor-Substituted Porphyrins. Angew. Chem. Int. Ed 2010, 49, 6646–6649. 10.1002/anie.201002118. 20687058

[ref102] Lu H.-P.; Tsai C.-Y.; Yen W.-N.; Hsieh C.-P.; Lee C.-W.; Yeh C.-Y.; Diau E. W.-G. Control of Dye Aggregation and Electron Injection for Highly Efficient Porphyrin Sensitizers Adsorbed on Semiconductor Films with Varying Ratios of Coadsorbate. J. Phys. Chem. C 2009, 113, 20990–20997. 10.1021/jp908100v.

[ref103] Tuerdi G.; Nizamidin P.; Kari N.; Yimit A.; Wang F. Optochemical properties of gas-phase protonated tetraphenylporphyrin investigated using an optical waveguide NH_3_ sensor. RSC Adv. 2018, 8, 5614–5621. 10.1039/C7RA11643H. 35542428 PMC9078192

[ref104] Maimaiti A.; Abdurahman R.; Kari N.; Ma Q.-r.; Wumaier K.; Nizamidin P.; Abliz S.; Yimit A. Highly sensitive optical waveguide sensor for SO_2_ and H_2_S detection in the parts-per-trillion regime using tetraaminophenyl porphyrin. J. Mod. Opt 2020, 67, 507–514. 10.1080/09500340.2020.1758817.

[ref105] Naik A.; Rubbiani R.; Gasser G.; Spingler B. Visible-Light-Induced Annihilation of Tumor Cells with Platinum–Porphyrin Conjugates. Angew. Chem. Int. Ed 2014, 126, 7058–7061. 10.1002/ange.201400533. 24852715

[ref106] Mahammed A.; Gross Z. Corroles as triplet photosensitizers. Coord. Chem. Rev. 2019, 379, 121–132. 10.1016/j.ccr.2017.08.028.

[ref107] Natale C. d.; Gros C. P.; Paolesse R. Corroles at work: a small macrocycle for great applications. Chem. Soc. Rev. 2022, 51, 1277–1335. 10.1039/D1CS00662B. 35037929

[ref108] Gallery J.; Gouterman M.; Callis J.; Khalil G.; McLachlan B.; Bell J. Luminescent thermometry for aerodynamic measurements. Rev. Sci. Instrum. 1994, 65, 712–720. 10.1063/1.1145090.

[ref109] Gouterman M. Oxygen Quenching of Luminescence of Pressure Sensitive Paint for Wind Tunnel Research. J. Chem. Educ. 1997, 74, 697–702. 10.1021/ed074p697.

